# Interfacial solvation pre-organizes the transition state of the oxygen evolution reaction

**DOI:** 10.1038/s41557-025-01932-7

**Published:** 2025-09-03

**Authors:** Ricardo Martínez-Hincapié, Janis Timoshenko, Timon Wagner, Eduardo Ortega, Jody Druce, Mariana C. O. Monteiro, Martina Rüscher, Joonbaek Jang, Elif Öykü Alagöz, Samuele Lasagna, Leon Jacobse, Arno Bergmann, Beatriz Roldan Cuenya, Sebastian Z. Oener

**Affiliations:** Department of Interface Science, Fritz-Haber Institute of the Max Planck Society, Berlin, Germany

**Keywords:** Electrocatalysis, Electrochemistry

## Abstract

The sluggish kinetics of the oxygen evolution reaction are an energetic bottleneck for green hydrogen production via water electrolysis. The reaction proceeds over a surface that undergoes (frustrated) phase transitions to accommodate bias-dependent excess charge. Here we perform Arrhenius analysis of common catalysts and correlate the activation energy and pre-exponential factor with the oxide’s structural adaptation via operando X-ray absorption spectroscopy and high-energy X-ray diffraction. We observe that the kinetics switch from a regime that is probably dominated by interfacial solvation to one where the surface energetics take over. This happens right at a transition potential between the α or β phases into the γ-crystal structure of Ni (oxy)hydroxides and when spectroscopic fingerprints of key intermediates emerge. Importantly, this turning potential is independent of the loading or the surface area and informs on the intrinsic catalyst activity. These results suggest that the catalyst activity is intrinsically linked to the initial interfacial solvation (pre-)step.

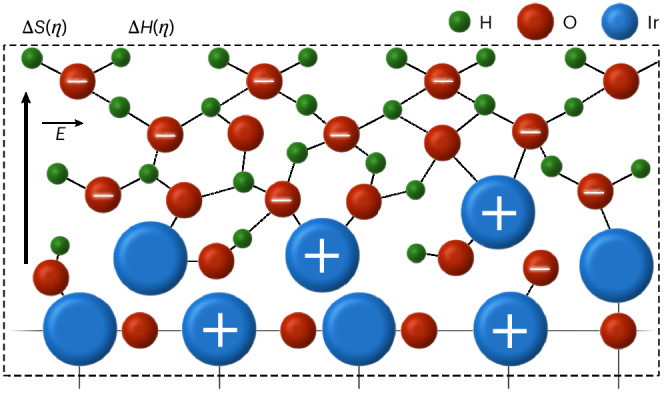

## Abstract (web summary)

The oxygen evolution reaction proceeds over a surface that undergoes (frustrated) phase transitions to accommodate bias-dependent excess charge. Now it has been shown that this excess charge is intimately linked to the interfacial solvation of ions and the pre-organization of the transition state, providing insight into intrinsic catalyst activities.

## Main

The oxygen evolution reaction (OER) is the key counter-reaction that balances the hydrogen evolution reaction, when electrochemically splitting water into green hydrogen and oxygen^1^. It occurs at (over)potentials at which the catalyst surface undergoes complete or frustrated structural and oxidation state changes that are associated with the generation of (oxy)hydroxide phases and charged intermediates^2^. Operando spectroscopy and microscopy have led to the understanding that OER-active centres are, in general, distinctly different from the pre-catalyst state, especially for the most active Ir-based^2^, Ni-based^3,4^ and Co-based^5–7^ materials. Such experimentally driven advances have increasingly challenged prevalent kinetic models and theory, especially with regard to the role of charged intermediates in the catalytic cycle.

Charged intermediates are generally associated with sluggish kinetics, such as those for the oxygen evolution^2,8^, oxygen reduction^9,10^ and ammonia oxidation reactions^11,12^. Density functional theory can provide information about the stability of such intermediates, including in the presence of an implicit or explicit solvent^13^. However, computational power is insufficient to capture long-range electric field effects inside the double layer, let alone the transition states^14^. Instead, it is widely assumed that catalyst activity in the inner sphere of heterogeneous interfaces adheres to outer-sphere principles. This includes the Bell–Evans–Polanyi relationship, which arose from homogeneous redox reactions^15–20^ and relates all activity differences to activation energy differences, while keeping the activation entropy constant. Furthermore, previous pictures have often argued in terms of a special chemical nature of charged intermediates^2,21^, neglecting the entropic and enthalpic effects of electric fields and, thus, a key component of interfacial electrochemistry. These assumptions are analogous to the ones done by many experimentalists.

Marcus–Hush-type and Butler–Volmer-type rate equations^22^ are widely used to analyse inner-sphere kinetics with linear Tafel slopes at a constant temperature, including for the OER^2^. In these outer-sphere frameworks, the bias impacts only the activation energy. Conversely, it is assumed that the ‘intrinsic’ activity is captured by the bias-independent exchange current density, *j*_0_. In this framework, bias-dependent changes in the double layer play a minor role, as long as the electron simply tunnels to the redox molecule in the outer sphere. Before electron transfer, water dipoles reorganize in the outer shell, which impacts only the activation enthalpy.

Experimentally, the (apparent) activation parameters governing the transition states can be accessed via Arrhenius analysis of temperature-dependent rates which provide the activation energy (*E*_A_) and the pre-exponential factor (*A*). *E*_A_ informs on the activation enthalpy (Δ*H* *=* *E*_A_ *–* *RT*) (where *R* is the gas constant and *T* the temperature) and *A* = exp(Δ*S* + Δ*S*_t_) on the activation entropy (Δ*S*) and thermal entropy (Δ*S*_t_), the latter containing the vibrational frequency factor. In contrast to biocatalysis or thermal catalysis, in electrocatalysis the free energy driving force of the catalyst is independently controlled by the external bias, whereas the rates can be tracked with very high precision. Conversely, temperature-dependent electrochemistry^23^ is a powerful tool to study the impact of the experimental control parameters on the transition states in electrocatalysis. For example, whereas many factors might impact the absolute values of the activation parameters, this approach allows to study shared fingerprints in the bias dependence of the activation energy and Arrhenius pre-exponential factor and relate them to bias-dependent changes on the surface or in the interfacial solvent.

**Fig. 1:**
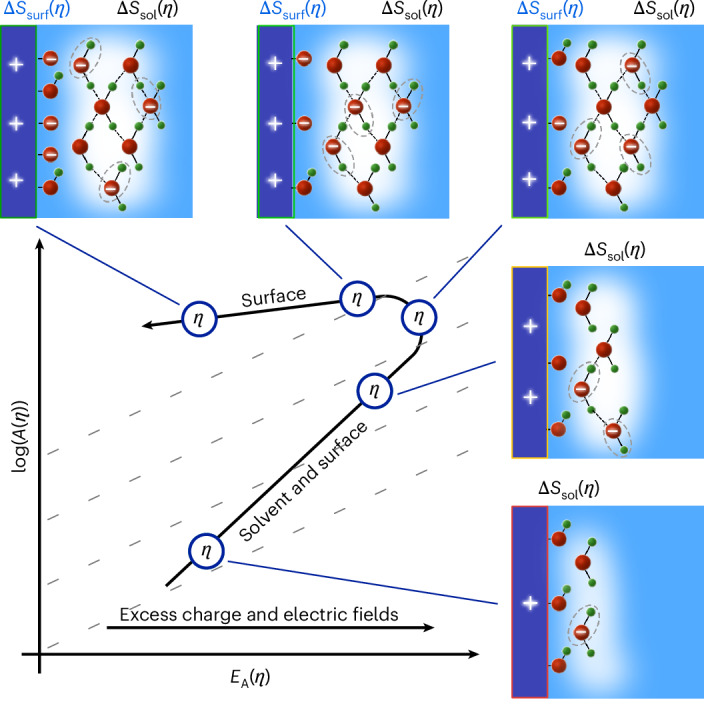
The impact of excess charge in the kinetic maps. The kinetic map depicts the bias-dependent *E*_A_(*η*) and log(*A*(*η*)). The dashed lines in the background represent isocurrent lines, that is, pairs of *E*_A_(*η*) and log(*A*(*η*)) that give rise to the same current density. Our results strongly suggest that bias-dependent excess charge and, thus, electric fields initially increase the activation energy, *E*_A_(*η*), and activation entropy, Δ*S*_sol_(*η*), of the solvation (pre-)step within the pre-exponential factor, log(*A*(*η*)). Eventually, the solvation pre-step becomes fast and kinetics accelerate because the bias reduces the activation energy directly. Therefore, the transition point is key for the overall kinetic response. Importantly, here, we are focussing on the bias-dependent changes of the activation parameters, whereas the absolute values might depend on many factors at the solid–liquid interface. The coloured frames (red, yellow and green) represent the bias-dependent electrode chemistry and structure. Adapted from our previous study^24^. Water with oxygen facing downward is based on recent spectroscopy (see below).

Recently, we performed Arrhenius analysis across reactions, spanning the hydrogen evolution^24^, oxygen reduction and ammonia oxidation reactions in alkali^25^ and even for purely ionically driven water dissociation and ion solvation in bipolar membranes^26^. For all of these, we observed conditions where *E*_A_(*η*) initially increases with bias and increasing rates are driven by an (over)compensating increase in log(*A*(*η*)) (where *η* is the overpotential; Fig. 1). These results led us to relate an increasing *E*_A_(*η*) with increasing excess charge, consistent with previous experiments^27^ and theory^28^. This excess charge induces electric fields that can impact Δ*S* in log(*A*(*η*))^29–32^. We hypothesized that increasing electric fields lead to an increasingly ordered (and connected^33^) hydrogen bond network, which could lead to ion delocalization in the transition state, which increases the number of microstates and, thus, the transition state entropy. In addition, other entropic contributions might also be changed^34,35^. Finally, once *E*_A_(*η*) and log(*A*(*η*)) have been maximized, the solvation pre-step becomes fast and the bias reduces *E*_A_(*η*), rapidly accelerating the rates. Thus, this transition point is critical for the kinetics but the specifics for the OER and the dependency on the local catalyst and solvent structure are still unknown.

More generally, our results on excess charge and ion delocalization resonate with findings in biocatalysis^36–38^, where strong electric fields at active sites of enzymes^39,40^ are thought to be critical to facilitate nucleophilic attack by water molecules or hydroxides in photosystem II^41,42^. In fact, the pre-organization of the active site into a spatially confined structure can be critical to reducing the penalty of conformational and solvent reorganizational motions^38,43^ of subsequent steps. To that end, (gated) side-chain deprotonation has been observed for the formation of the charged Mn(IV)-O^•^ intermediate during photosynthetic water splitting^44^, giving hints into how enzymes manage uncompensated charge. Beyond biocatalysis, electric fields might even be present in thermal catalysis on chemically polarized catalyst surfaces, let alone in electrochemically driven thermochemical reactions^45,46^. Thus, the role of excess charge is of fundamental relevance across the field of heterogeneous catalysis.

Here we link the bias-dependent activation energy and pre-exponential factor with the formation of the active electrocatalyst state via operando X-ray diffraction (XRD) and operando X-ray absorption spectroscopy (XAS). We identified a critical turning potential at which the bias-dependent kinetics switch from a regime that is dominated by interfacial solvation to one where the surface energetics become more dominant in the bias-dependent changes of the activation parameters. At this critical turning point, spectroscopic fingerprints of key-charged OER intermediates emerge and recent optical spectroscopy observes bias-dependent ordering of the hydrogen bond network.

## Entropy–enthalpy relationships for OER

The (apparent) activation energy (*E*_A_) and pre-exponential factor (log(*A*)) were obtained in both alkaline (0.1 M Fe-purified and Fe-containing KOH) and acidic (0.1 M H_2_SO_4_) electrolytes via chronoamperometry at different overpotentials and temperatures using Ni(OH)_2_ or NiFe LDH (layered double hydroxide) nanoparticles in alkaline media and IrO_*x*_ nanoparticles in acidic media. These catalysts are among the most widely studied OER catalysts in the literature^1^, due to their relatively high stability^47^ and activity, which is typically related to different degrees of crystallinity, domain sizes and oxyhydroxide phases. In addition, iron incorporation has been shown to be a critical factor in influencing the catalyst activity^4,48,49^ and therefore needs to be controlled for by catalyst and electrolyte preparation protocols and during operation^50^, including in industrial alkaline water electrolysis and proton exchange membrane electrolysers in acidic media^1,51^. Therefore, understanding the fundamental activity and stability limitations of these catalysts has been a central research topic for decades.

**Fig. 2:**
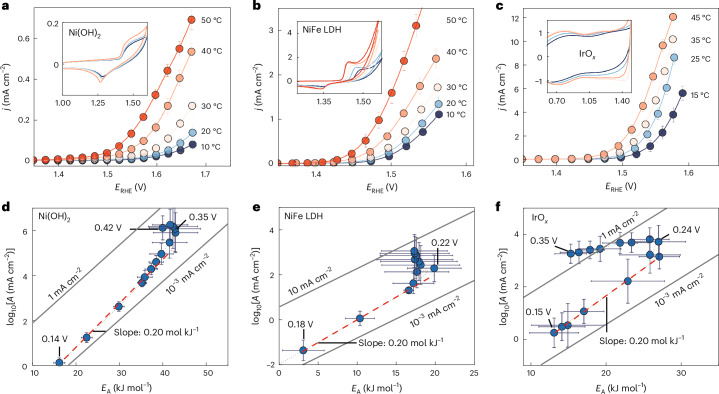
Polarization curves and overpotential dependence of the *E*_A_(*η*) and log(*A*(*η*)). **a**–**c**, Characteristic polarization curves for Ni(OH)_2_ (**a**) and NiFe LDH (**b**) in 0.1 M Fe-purified KOH and IrO_*x*_ (**c**) in 0.1 M H_2_SO_4_. **d**–**f**, Overpotential-dependent log(*A*(*η*)) versus *E*_A_(*η*) for Ni(OH)_2_ (**d**), NiFe LDH (**e**) and IrO_*x*_ (**f**) from **a**–**c**, respectively. Contrary to common belief, the bias-dependent kinetics are not limited by one process, but cascade through regimes where the kinetics are probably dominated by interfacial solvation until a turning or transition potential has been reached, where the kinetics appear to be dominated by the energetics of surface intermediates. The lower the turning (over)potential between the two regimes, the higher the intrinsic catalyst activity. For all measurements, multistep chronoamperometry was performed with individual potential hold for at least 60 s. The overpotentials are corrected for the ohmic IR (current–resistance) drop and the temperature-dependent equilibrium potential of the OER. For uncorrected potentials, see Supplementary Fig. 10. The grey diagonal lines in the background are isocurrent lines at 25 °C, that is, pairs of log(*A*) and *E*_A_ that result in the same current. Values are means and error bars reflect the s.d. for *E*_A_ (slope) and *A* (intercept) from Arrhenius analysis, based on five temperatures. The dashed lines show the fits to extract the compensation slopes, whereas short dotted lines are guides to the eye. Source data

Ex situ electron microscopy (Supplementary Figs. 1–5) indicates that the overall structural integrity of both Ni-based and Ir-based catalysts is maintained before and after the reaction. Scanning transmission electron microscopy (STEM) and energy-dispersive X-ray spectroscopy (EDS) of selected IrO_*x*_ samples did not show any substantial compositional changes before and after the reaction (Supplementary Table 1). Representative polarization curves for all materials are shown in Fig. 2a–c. For all measurements, the steady-state current was measured with individual potential holds of 60 s to minimize the impact of pseudo-capacitive (dis)charging current (Supplementary Figs. 6 and 7). All bias-dependent Arrhenius curves (log(*j*) versus *T*^−1^), where *j* is the current density, were fitted with linear slopes, with most data exhibiting linear regression *R*^2^ values well above 0.95 (Supplementary Figs. 8 and 9). High linear regression *R*^2^ values are important, because the current is interpolated from the experimental range to infinite temperatures to obtain log(*A*). Here we use rotating disc electrodes and limit the geometric current densities to <10 mA cm^−2^_geo_ to avoid any substantial impact of mass transport. Finally, all overpotentials have been corrected for the temperature dependence of the equilibrium potential of the OER half-reaction^52^ (Methods).

Figure 2d–f shows the kinetic maps (*E*_A_ versus log(*A*)) for Ni(OH)_2_ and NiFe LDH in alkali and IrO_*x*_ in acid, respectively. Supplementary Fig. 10 shows the OER kinetics without the correction of the temperature-dependent equilibrium potential. It is noteworthy that all OER catalysts display very similar bias-dependent changes in the activation parameters, which principally differ by the potential at which the maximum activation energy and pre-exponential factor are reached and their absolute values. Starting at low overpotentials, both *E*_A_ and *A* rise together with bias. In this regime, increasing rates are driven by an increasing pre-exponential factor, which overcompensates the increasing activation energy. We have previously observed very similar compensation effects across electrocatalytic interfaces^24–26^, including in bipolar membrane junctions that are purely ionically driven. Conversely, we assign these initial changes in the pre-exponential factor primarily to changes in the interfacial solvent network that accelerate the interfacial solvation (pre-)step^24–26^.

After the initial rise, the activation energy and pre-exponential factor are maximized at an overpotential of 0.35 V, 0.22 V and 0.24 V for Ni(OH)_2_, NiFe LDH and IrO_*x*_, respectively, in line with the much lower activity of Ni(OH)_2_, compared with NiFe and IrO_*x*_. Importantly, the potential of maximum activation energy and pre-exponential factor are essentially independent of the normalization by the electrochemically active surface area (ECSA) (Supplementary Fig. 11) or the catalyst loading (Supplementary Fig. 12). Instead, it reflects the intrinsic catalyst activity and is very close to the potential where key intermediates emerge in the spectroscopic data. The sooner that this point is reached the faster the kinetics switch from a slow (pre-)region dominated by interfacial solvation into a fast ‘Butler–Volmer’ regime, where the decreasing activation energy rapidly increases the rates (compare Fig. 2a–c with Fig. 2d–f). Compared with the overpotential defined at any arbitrary current density, which scales with catalyst loading, or the onset potential, which has no rigorous definition whatsoever, this potential is well defined as the point where log(*A*) and *E*_A_ are maximized.

At potentials beyond the transition point, we observed that the activation energy starts to decrease with bias, resembling Butler–Volmer kinetics, which are dominated by adiabatic electron transfer, albeit with deviations, which could be caused by bias-dependent coverage of intermediates, the completion of the frustrated phase transition (overoxidation) or the extension of the reaction zone into the bulk^53,54^. Finally, the rapidly increasing currents beyond the transition potential quickly lead to mass transport limitations in liquid electrolyte cells. Even if correction of the potential for the ohmic resistance does not impact our results in any substantial way (Supplementary Fig. 13), bubble formation^55^ could change the number of available active sites, which could drastically impact the kinetics.

**Fig. 3:**
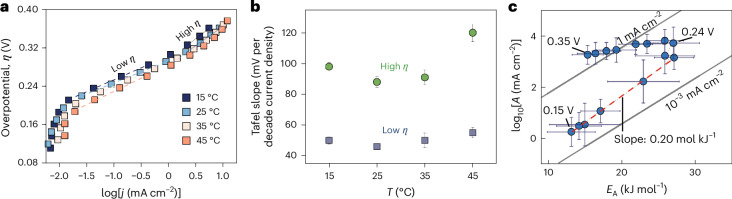
The limitations of contemporary Tafel analysis. The analysis of linear Tafel slopes precludes the accurate analysis of bias-dependent charge transfer coefficients (*α*). **a**, Two regions with seemingly linear Tafel slopes for IrO_*x*_ nanoparticles in Fig. 2f. **b**, Temperature (*T*) dependence of the Tafel slopes $b=2.3RT/F\left({\alpha }_{{\mathrm{H}}}+T{\alpha }_{{\mathrm{S}}}\right)$, which informs on the entropic (*α*_S_) and enthalpic contributions (*α*_H_) of the charge transfer coefficient $\alpha ={\alpha }_{{\mathrm{H}}}+T{\alpha }_{{\mathrm{S}}}$ with the gas and Faraday constants *R* and *F*, respectively. Values are means and error bars reflect the s.d. from fits of linear Tafel slopes in **a**. **c**, Full bias-dependent kinetics with the key compensation region at low bias and a turning overpotential around 0.24 V (1.47 *V*_RHE_), which are not apparent from **a** and **b**, due to the neglect of the bias dependence of the charge transfer coefficient. Values are means and error bars reflect the s.d. for *E*_A_ (slope) and *A* (intercept) from the Arrhenius analysis, based on five observations. The dashed lines show fits to extract the respective slopes. Overpotentials are corrected for the temperature dependence of the equilibrium potential. Source data

In contemporary studies, the turning potential is obscured when analysing seemingly linear Tafel slopes at a constant temperature. Figure 3a shows a typical log(*j*) versus *E* plot for the IrO_x_ nanoparticles with two linear Tafel fragments. The underlying assumption in conventional Tafel analysis is always that there is an ‘intrinsic activity’, designated by the exchange current density, *j*_0_, and the activation energy is then simply reduced with bias, that is, the kinetics follow Butler–Volmer-type rate equations and are free of mass transport or double-layer effects^56,57^. This outer-sphere approach neglects the temperature (*T*) and overpotential (*η*) dependence of the charge transfer coefficient, $\alpha \left(\eta ,T\right)={\alpha }_{{\mathrm{H}}}\left(\eta \right)+T{\alpha }_{{\mathrm{S}}}(\eta )$, where *α*_H_ and *α*_S_ designate the enthalpic and entropic parts that directly relate to $\Delta H(\eta )$ and $\Delta S(\eta )$, respectively^11,29,58^. Figure 3b shows the temperature dependence of the two regimes in Fig. 3a, which is related to *α* via $b=2.303{RT}{\left(F\alpha \left(\eta ,T\right)\right)}^{-1}$ (ref. ^58^), with Faraday’s constant *F*. The initial slope has a higher entropic contribution (lower temperature dependence), whereas, at higher bias, the enthalpic contribution dominates (stronger temperature dependence). Figure 3c shows the actual kinetics. Clearly, the turning point is obscured in Fig. 3a,b, due to the bias-dependent *α*(*η*), which is neglected when fitting linear Tafel fragments to continuously changing log(*j*) versus *E* curves.

## Fingerprints of intrinsic activity in the kinetic maps

The kinetic maps in Fig. 2d–f of nominally different materials are strikingly similar. This finding resonates with the notion that different OER pre-catalysts are forced into a few prevailing and active oxyhydroxide or amorphous oxide phases under operation^1^, fundamentally limiting OER activity across materials and pH. Fe incorporation into NiO_*x*_-based catalysts^4,48^ and the role of the crystallinity for IrO_*x*_ catalysts^2,59^ are among the two most widely studied factors that have been proposed to affect the intrinsic OER activity. However, even for these cases, ongoing debates about the impact of the number of active sites and their evolution versus intrinsic activity exist in the literature^60^. Therefore, we explored some of these cases to test whether the intrinsic activity is reflected in distinct differences in the kinetic maps.

**Fig. 4:**
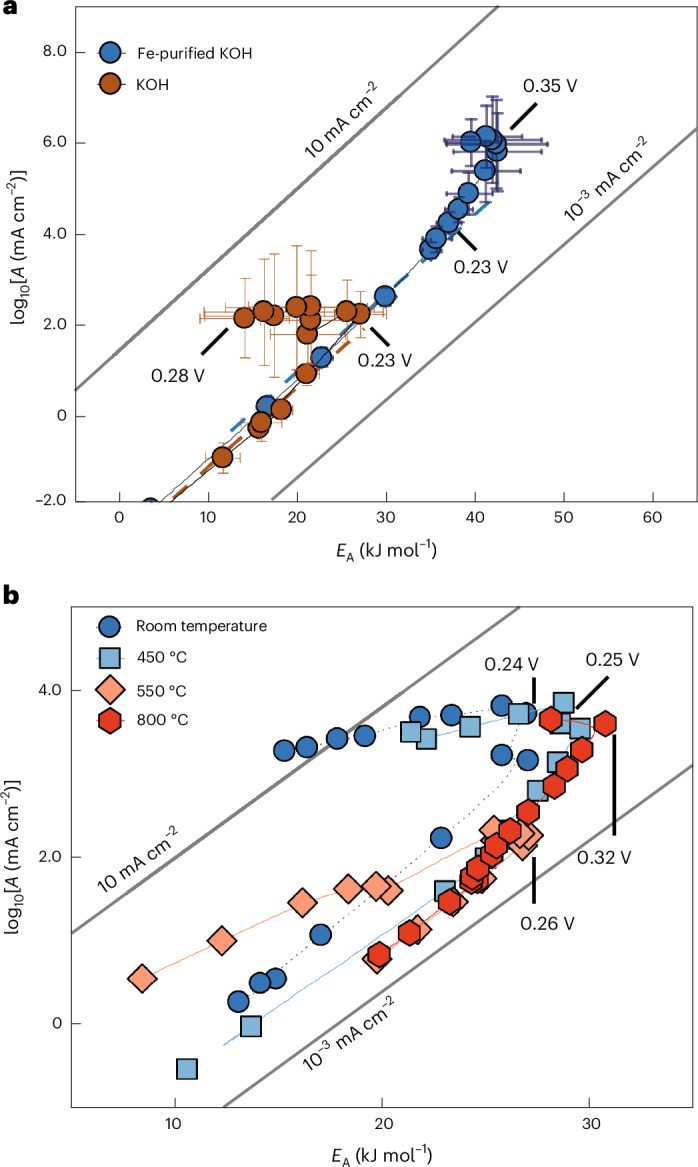
Bias-dependent *E*_A_(*η*) and the log(*A*(*η*)) for different OER catalysts. The turning potential between the slow compensation and the fast Butler–Volmer regime is critical to understand the intrinsic catalyst activity. **a**, Kinetic map (log(*A*(*η*)) versus *E*_A_(*η*)) for Ni(OH)_2_ in Fe-purified and Fe-containing 0.1 M KOH. **b**, Kinetic map of IrO_*x*_ in 0.1 M H_2_SO_4_ with different calcination temperatures during the pre-treatment and, thus, degrees of crystallinity. Increasing calcination temperatures lead to increasing crystallinity, where IrO_*x*_ at room temperature is highly amorphous. In all cases, the kinetics are similar and principally differ in the turning potential where the activation energy and pre-exponential factor are maximized. Values are means and error bars reflect the s.d. for *E*_A_ (slope) and *A* (intercept) from Arrhenius analysis, based on five observations. Overpotentials are corrected for the IR drop and the temperature-dependent equilibrium potential. Some error bars are omitted for clarity, but shown in Supplementary Fig. 16. The short dotted lines are guides to the eye. Source data

We examined the impact of iron incorporation in the kinetics using Ni(OH)_2_ in contact with Fe-free (pre-purified) and Fe-containing KOH solutions (Fig. 4a). Indeed, we observed that the transition point in the kinetic plot shifts from an overpotential of 0.35 V to 0.25 V, going from the Fe-free to the Fe-containing KOH solution, although the compensation slopes before the transition potential remains essentially the same. Previously, the enhanced activity was ascribed to a higher bulk electrical conductivity and, among other propositions, a participation of the Fe at the active site, for example, the reaction centre Ni-O-Fe might stabilize OER intermediates more efficiently than the Ni-O-Ni centre^4,48,61^ or the presence of cooperative Fe sites in FeO_*x*_ clusters^3^. Furthermore, Fe appears to impact the thickness of the oxyhydroxide layer^62,63^. Clearly, our results support an intrinsic impact of Fe decoration on a Ni oxyhydroxide host, because an increase in the number of sites alone would not lead to a changing turning potential. Although the Ni(OH)_2_ activity with the Fe-containing KOH shows a lower *E*_*A*_ than the Fe-free KOH, the log(*A*) is, in fact, lower by two orders of magnitude as well. The impact of the different turning potential has a larger impact, as will become more evident in the following.

Figure 4b shows kinetic maps for IrO_*x*_ nanoparticles that were calcined at different temperatures to control the degree of crystallinity, as evidenced in the voltammograms and XRD patterns (Supplementary Figs. 14 and 15). The as-received iridium oxide catalyst is denoted as amorphous, because only diffraction peaks from the minor metallic Ir phase are observed in the XRD patterns. This suggests that the oxidized fraction of the material lacks long-range order and is therefore designated as X-ray amorphous IrO_x_. Detailed Rietveld analysis of the samples calcined at temperatures ≥350 °C shows a systematic increase in the crystallite size of the majority (≥95 wt%) IrO_2_ and minority Ir (3–4 wt%) phase with increasing temperatures (Supplementary Fig. 15 and Supplementary Table 2). Furthermore, ex situ TEM (Supplementary Figs. 1 and 2) reveals progressive crystallization of the IrO_*x*_ catalysts on annealing, with surface crystallinity becoming slightly more pronounced after the reaction (Supplementary Fig. 4). Samples annealed at 250 °C and 350 °C remain largely amorphous, although small crystalline domains can be identified, suggesting the initial stages of ordering.

Strikingly, the different activity between the different calcinated samples is not reflected in the absolute *E*_A_(*η*) and log(*A*(*η*)), which trace along almost identical values with bias, but in the transition point, which shifts to more positive overpotentials with decreasing activity and increasing calcination temperature and crystallinity. For IrO_*x*_, the turning overpotential is 0.24 V and it slowly rises to 0.32 V when calcined at 250 °C, 350 °C, 450 °C, 550 °C and 800 °C, respectively (for the whole range, see Supplementary Fig. 16). These results suggest that the degrees of IrO_*x*_ crystallinity change not only the number of active sites, but also the intrinsic activity by changing the oxygen coordination environment.

The kinetic maps in Figs. 2–4 identify the turning point as a critical potential for the OER and possibly more broadly across electrocatalysis in aqueous solvents. Our results strongly point toward a fundamental limitation for anodic oxide catalysts in aqueous solvents and might help elucidate why, despite decades of research, the most active catalysts have remained essentially the same materials. Even for NiFe LDH and IrO_*x*_ nanoparticles, the kinetics only transition into a Butler–Volmer region at an overpotential of ~250 mV. Previously, OER activity limitations have been linked to the need to generate and stabilize charged intermediates in metastable oxide structures. Therefore, we explored how these intermediates are linked to the kinetic maps.

## Fingerprints of key intermediates in the entropy–enthalpy relationships

Incomplete (frustrated) oxidation state changes and the related charged intermediates have been detected with operando spectroscopy on Ir-based^2,8,64^, Ni-based^4,48,61^ and Co-based^7,8^ catalysts. It was argued^41,42,65^ that the excess charge is needed for nucleophilic attack by water molecules or hydroxides in analogy to water oxidation in photosystem II^41,42^. However, so far, the direct impact on the transition state of the OER has remained opaque and adiabatic rate equations and theory were employed to rationalize the impact of excess charge in the whole potential range^2,66^.

**Fig. 5:**
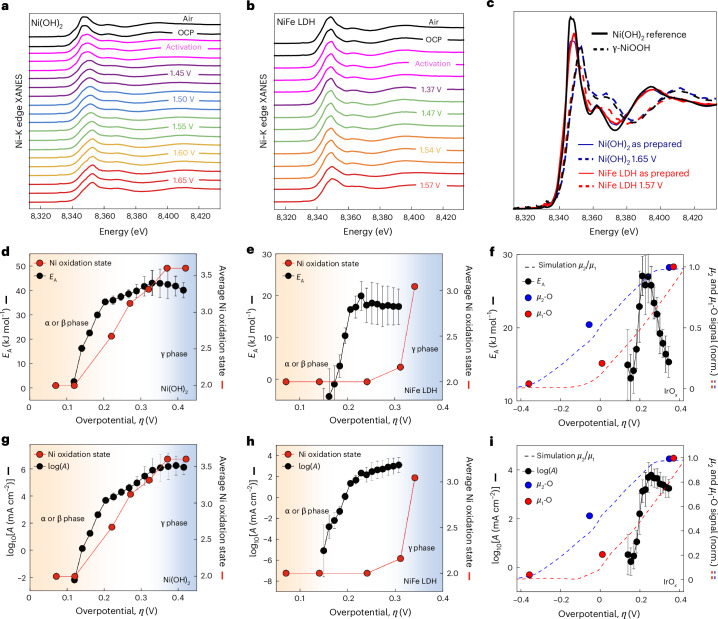
Fingerprints of key intermediates in the entropy–enthalpy relationships. The switch in the kinetic regime occurs in the transition region between different crystal phases, which are related to the emergence of charged oxidation states. **a**–**c**, Potential-dependent XANES at selected potentials, starting at open circuit potential (OCP), for Ni(OH)_2_ (**a**) and NiFe LDH (**b**), as well as comparison of in situ XANES with the reference spectra (**c**). **d**,**e**, Bias-dependent activation energy, *E*_A_(*η*), and average Ni oxidation state for Ni(OH)_2_ in Fe-purified 0.1 M KOH (**d**) and NiFe LDH in 0.1 M KOH (**e**). **f**, *E*_A_(*η*) and normalized *µ*_1_-O and *µ*_2_-O signals for IrO_*x*_ in 0.1 M HClO_4_. **g**,**h**, Bias-dependent pre-exponential factor, log(*A*(*η*)), and average Ni oxidation state for Ni(OH)_2_ in Fe-purified 0.1 M KOH (**g**) and NiFe LDH in 0.1 M KOH (**h**). **i**, The log(*A*(*η*)) and normalized (norm.) *µ*_1_-O and *µ*_2_-O signals for IrO_*x*_ in 0.1 M HClO_4_. The shaded background colours in **d**, **e**, **g** and **h** designate the transition between the αβ and γ crystal structures as extracted from XRD (Supplementary Figs. 21–24). Values of activation parameters are means and error bars reflect the s.d. for *E*_A_ (slope) and *A* (intercept) from the Arrhenius analysis, based on five observations (temperatures). The assignment of the (average) oxidation states (Ni^2+^, Ni^3.6+^, *µ*_1_-O, *µ*_2_-O) follows documented protocols for the analysis of operando XRS. The *µ*_1_-O and *µ*_2_-O data are from ref. ^67^. The dashed blue and red lines in **f** and **i** indicate simulations of *µ*_2_ and *µ*_1_ species from ref. ^67^. All kinetic data were extracted from a temperature-controlled, rotating disc electrode set-up. Therefore, small potential shifts might be present compared with the data from the operando cells. Overpotentials are corrected for the temperature dependence of the equilibrium potential. Source data

Operando XAS for Ni(OH)_2_ and NiFe LDH was performed by changing the bias in a step-wise manner and collecting Ni–K edge X-ray absorption near-edge spectra (XANES) (Fig. 5a–c). The presence of isosbestic points in normalized XANES (Supplementary Fig. 17) suggests that variations between different spectra can be assigned to a change in a single latent variable, which in this case we can associate with the average oxidation state of Ni. Conversely, we calculate the oxidation state of Ni in our catalyst as a weighted average: $2\times w\left[{\mathrm{N{i}}}^{2+}\right]+3.6\times w\left[{\mathrm{N{i}}}^{3.6+}\right]$, where $w\left[{\mathrm{N{i}}}^{2+}\right]$ and $w\left[{\mathrm{N{i}}}^{3.6+}\right]$ are the concentrations of the respective species, as obtained from linear combination analysis (LCA)-XANES (Methods and Supplementary Figs. 18 and 19). We emphasize, however, that our analysis does not exclude the presence of possible intermediates, but merely assumes that the difference between the XANES for all species involved linear changes with the respective Ni oxidation state.

Analogously, operando high-energy XRD (HE-XRD) experiments were performed to follow the bulk, structural potential-dependent adaptations (Methods) and the formation of the γ-NiOOH_*x*_ phase—supposedly the predominant phase linked to OER activity. The changes in the catalyst oxidation state (tracked by XAS) and structure (as probed by XRD) can then be linked to the *E*_*A*_–log(*A*) kinetic maps. We note that both operando XAS and HE-XRD are bulk sensitive and only indirect information about the surface intermediates can be extracted. However, by linking it to surface-sensitive electrochemistry, especially the kinetic maps, we can link the spectroscopy to the OER transition state.

Figure 5 shows the correlations of the concentration of the key (oxyhydr)oxide species identified through operando XAS at different overpotentials with *E*_A_(*η*) (Fig. 5d–f) and log(*A*(*η*)) (Fig. 5g–i). For Ni(OH)_2_, both *E*_A_(*η*) (Fig. 5d) and log(*A*(*η*)) (Fig. 5g) rise and saturate in a manner that very closely mirrors the potential-dependent increase in the Ni oxidation state in the spectroscopic data. Such a charged state of the near-surface oxygen chemistry can be related to the electrophilic oxygen sites^1^. For NiFe LDH, *E*_A_(*η*) (Fig. 5e) and log(*A*(*η*)) (Fig. 5h) rise and saturate earlier, just before the increase in Ni oxidation state. This indicates that NiFe LDH is able to stabilize excess charge at much lower potentials than Ni(OH)_2_. With further increasing Ni oxidation, *E*_A_(*η*) starts to decrease. One should note, however, that, in a few cases, we observed small further structural or chemical changes in our XAS data, even after three consecutive scans (approximately 15 min; Supplementary Fig. 20). Thus, although Fig. 5 correlates the steady-state Faradaic OER current with the spectroscopic fingerprints, the obtained spectroscopy can be affected by the slow kinetics of analysed catalyst transformations.

Finally, operando XRD provides information on the transformation of α or β phases into the γ phase, and the results match remarkably well with the transition point in the kinetics, as indicated by the orange and blue shaded background in Fig. 5d,e,g,h and Supplementary Figs. 21 and 24. We hypothesized that this reflects the stabilization of excess charge in the frustrated phase transition between these two dissimilar oxide phases. For IrO_*x*_, we find that *E*_A_(*η*) (Fig. 5f) and log(*A*(*η*)) (Fig. 5i) are maximized at the potentials where the *μ*_2_-O concentration saturates, in line with previous results^67^ indicating that these charged oxide species are key for the OER. The *µ*_2_-O species reflect the bridging O sites, whereas the *µ*_1_-O sites are linked to Ir_cus_, the coordinatively unsaturated sites (cus), for example, on the IrO_2_(110) surface. It is believed that the cus sites are O_2_-forming sites whereas O_br_ interacts with protons and, thus, the interfacial water network^2,67^.

## Discussion

Our results are critical to understanding the very nature of the overpotential and the impact of excess charge in electrocatalysis. Across different OER catalysts and different reactions^24,25^, and even in bipolar membranes^26^, we observed that the bias can charge up the interface, inducing entropy–enthalpy compensation, where log(*A*(*η*)) and *E*_A_(*η*) increase together. When the surface becomes polarizable, outer-sphere-type theory and experimental methods have run their course, because they do not capture the kinetic effects of bias-dependent excess charge and electric fields inside the double layer or in the structure of flexible oxide networks. These can impact the shared bond-energy factors in the partition functions that give rise to both Δ*H* and Δ*S* (ref. ^68^). Only once *E*_A_(*η*) and log(*A*(*η*)) have been maximized and the initial solvation pre-step is fast enough can the bias directly reduce the activation energy. This happens because the excess charge density is stabilized in the form of charged intermediates and/or the active catalyst state possesses distinct interaction with solvated ions. Here we discover that this transition point is key for the intrinsic catalyst activity and independent of the catalyst loading or surface area.

We have previously identified the entropy–enthalpy compensation slopes, where *E*_A_(*η*) and log(*A*(*η*)) increase together, as a means to detect and study excess charge under bias^24–26^. For metallic surfaces, we have observed higher slopes, such as for Au during the hydrogen evolution reaction^24^ in acid (~0.37 mol kJ^−1^) or base (~0.23 mol kJ^−1^). On the other hand, for ammonia oxidation and oxygen reduction^25^, we observed similar values compared with here^25^ (~0.19–0.2 mol kJ^−1^), whereas we detected the lowest values (~0.18 mol kJ^−1^) for the pseudo-capacitive Pt oxidation in alkali^25^ (${{\mathrm{Pt}}}-{{\mathrm{OH}}}+{{{e}}}^{-}\leftrightarrow {{\mathrm{Pt}}}+{\mathrm{O{H}}}^{-}$). Thus, we hypothesize that the different compensation slopes reflect differences in the build-up of excess charge. Compared with a metallic surface, pseudo-capacitive oxidation state changes store some of the charge density in the formation of new charge-compensated covalent bonds. Furthermore, for pronounced pseudo-capacitive processes, we expect an increasing contribution of surface configurational entropy. To separate these further in the future, the bias-dependent activation parameters can be correlated with electrochemical methods, such as bias-dependent, double-layer capacitance^26^ or potential jumps^25^ to study time-dependent aspects of charge accumulation. Finally, optical spectroscopy and X-ray spectroscopy (XRS) on the interfacial solvent at reactive interfaces, such as XRD, FT-IR, Raman or sum frequency generation, will be critical to inform on the bias-dependent, molecular double-layer structure^69–71^. In fact, during the review of this manuscript, a key spectroscopy study on the interfacial solvent was published^72^.

**Fig. 6:**
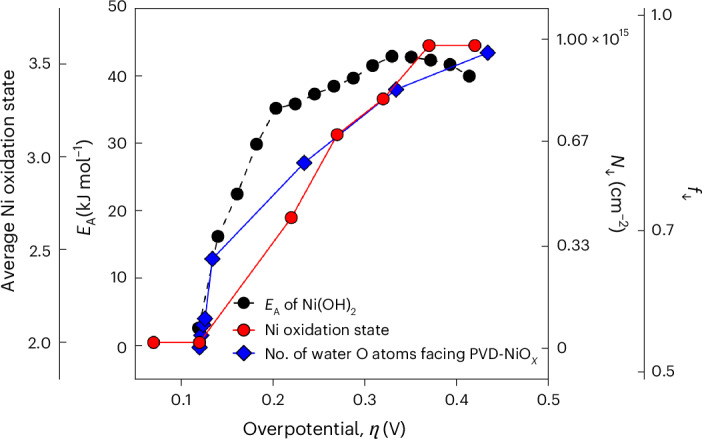
Kinetic impact of interfacial solvent ordering. Bias-dependent excess charge impacts the kinetics and the chemistry and structure on the surface and the solvent. Shown are bias-dependent activation energy, *E*_A_(*η*), and average Ni oxidation state for Ni(OH)_2_ from our study and density of ordered water, with O atoms facing downward (downward-facing arrow) from ref. ^72^ for NiO_*x*_ prepared by physical vapour deposition (PVD). The results are in striking agreement with the overpotential where log(*A*(*η*)) and *E*_A_(*η*) are maximized (kinetics), the local chemical potentials are changed (XRS) and the water layer ordering is saturating (SHG). *f*, monolayer fraction net-aligned. Source data

Speelman et al. used second harmonic generation (SHG) to study bias-dependent water ordering inside the compact Stern layer on a (physical vapour-deposited) NiO_*x*_ electrode^72^. Their findings strongly support our hypothesis on the impact of the solvent. Figure 6 combines our results for Ni(OH)_2_ in 0.1 M KOH, pH 13, with those of Speelman et al. of the physical vapour-deposited NiO_*x*_ electrode^72^ in 1 M NaClO_4_, pH 13. Despite the different conditions, we found a qualitative and quantitative match of the overpotential where the (apparent) activation energy and pre-exponential factor are maximized, the local chemical potentials on the catalyst surface are changed and the Stern water layer saturates. This combined work highlights the electrostatically coupled nature of the interface between the catalyst and solvent and the importance of not neglecting one over the other in kinetic pictures.

Our results across reactions^24–26^ strongly suggest that the results by Speelman et al.^72^ are not limited to the OER, which were analysed with temperature-independent Tafel slopes. In contrast, the SHG results are probably much more broadly relevant for the entropic and enthalpic changes in an increasingly ordered and possibly connected^33^ hydrogen bond network. Using a simple Ising model, Speelman et al. estimated that the work to reorient the interfacial water molecules was around ~80 kJ mol^−1^ for NiO_*x*_, whereas our results show a maximum *E*_A_(*η*) of about 40 kJ mol^−1^. Noteworthy, for Fe_2_O_3_, Speelman et al.^73^ found a work of about 40–50 kJ mol^−1^. Clearly, further temperature-dependent electrochemistry in combination with optical spectroscopy and XRS is needed to decipher the impact of the oxidation state changes, the role of electrolyte ions and the hydrogen bond network on the bias-dependent activation parameters.

In light of our results, one might ask the question why excess charge is even evolved in enzymes, because it appears to be linked to entropy–enthalpy compensation in aqueous media. Recent results suggest that enzymes pre-organize the solvation environment, possibly without accruing a large enthalpic penalty^36–38^ and even using gated side-chain deprotonation^44^. Conversely, to improve synthetic catalysts, including those used for the OER, design and control of the liquid solvation environment, possibly in an inorganic interphase sandwiched between solid and liquid, might be the only way to overcome historical limitations. Finally, our reasoning around the compensation at solid–liquid interfaces might be extendable to polarization and electric fields in thermal catalysis. For any (electro)chemical polarization of a catalyst, the dielectric space between the uncompensated charges might provide an environment to pre-organize the transition states and increase the activation entropy, whether bonds need to be dissociated or formed or ions (de)solvated.

## Methods

### Cell preparation and cleaning procedures

All electrochemical cells and associated glassware were cleaned by immersion in an acidic potassium permanganate solution and left overnight. The glassware was rinsed with a diluted piranha solution and boiled several times in ultrapure Milli-Q water (resistivity ≥18.2 MΩ cm). Plastic components, including polymer-based electrochemical cells and containers, were cleaned by immersion in a 10% (v/v) sulfuric acid solution overnight, followed by repeated rinsing with Milli-Q water. The effectiveness of the cleaning procedure was verified by cyclic voltammetry using a platinum wire as the working electrode. This test also ensured proper functioning of the reference electrode.

### Purification of KOH electrolyte

KOH was purified to remove trace metal contaminants following a protocol from Trotochaud et al.^4^. Semiconductor-grade KOH pellets (99.99%) were dissolved to prepare 1 M KOH. In an inert atmosphere glovebox, ~2 g of Ni(NO_3_)_2_·6H_2_O was dissolved in Milli-Q water (resistivity ≥18.2 MΩ cm) and mixed with 1 M KOH, forming a Ni(OH)_2_ precipitate. The mixture was centrifuged at 9,000 rpm for 1 h and the supernatant discarded. The precipitate was washed 3× with Milli-Q water and small additions of 1 M KOH, followed by centrifugation at 9,000 rpm after each wash. The solid was redispersed in 50 ml of 1 M KOH, stirred for at least 10 min and left to equilibrate overnight. The suspension was centrifuged (9,000 rpm, 1 h) and the supernatant collected. After two additional rest and centrifugation steps, any residual Ni(OH)_2_ was removed. The purified 1 M KOH stock solution was diluted tenfold to obtain 0.1 M KOH.

### Electrochemical set-up

The electrochemical experiments were performed using a rotating disc electrode set-up in a glass cell (for acidic media) or a plastic cell (for alkaline conditions), with a PINE rotator and a Gamry Reference 3000 potentiostat. A platinum wire served as the counter-electrode and a GASKATEL reversible hydrogen electrode (RHE) was used as the reference electrode. The working electrode was prepared by dropping 10 μl of the catalyst ink on to a previously polished and cleaned, glassy carbon rotating disc electrode (area: 0.196 cm², PINE). The Ir-based catalyst ink was prepared by dissolving 5 mg of IrO_*x*_ (Premion 99.99%, Alfa Aesar) in 3,980 μl of ultrapure water (resistivity 18.2 MΩ cm, total organic carbon <1 parts per billion (ppb)), 1,000 μl of isopropanol and 20 μl of Nafion solution (5 wt%). For alkaline media, the catalyst inks were prepared by dissolving 2 mg of catalyst (commercial NiOH_2_ or synthesized NiFe LDH following previous protocols^74^) in 200 μl of ultrapure water, 800 μl of isopropanol and 5 μl of PiperION (10 wt%, Versogen). In both cases, the inks were horn sonicated for 20 min. The electrolytes were prepared using sulfuric acid diluted in ultrapure water (resistivity 18.2 MΩ cm and total organic carbon <1 ppb). For the preparation of the 0.1 mol l^−1^ of KOH solution, an additional purification procedure was followed to obtain an Fe-free electrolyte. Before any measurements were taken, the electrolytes were purged with Ar for 15 min to remove dissolved oxygen and the gas was kept over the solution during the experiments to prevent oxygen intrusion.

All experiments were conducted under an Ar atmosphere with the electrode rotating at 1,600 rpm to minimize mass transport limitations. Despite this, bubble accumulation at the electrode surface occasionally occurred. A fresh thin film of catalyst (Ni(OH)_2_ or NiFe LDH) was drop cast for the individual measurements at each temperature. Electrolyte temperatures ranged from 10 °C to 50 °C, controlled by a Julabo Presto A85 and monitored using a Teflon-coated thermocouple (also cleaned with the KMnO_4_-based procedure). Measurements >50 °C were avoided to limit excessive evaporation. Chronoamperometry was conducted with individual potential holds for a least 60 s, where the reported current value represents the average of the last 300 points collected.

### Equilibrium potential temperature correction

The equilibrium potential of the OER was calculated at each temperature taking into account the temperature sensitivity (0.8 mV K^−1^) of the redox potential of the OER half-reaction^52^. Overpotentials were calculated by subtracting the equilibrium potentials at each temperature from the IR-corrected applied potentials. A set of evenly spaced overpotentials across temperatures was obtained using linear interpolation. Corrected currents were interpolated from averaged steady-state currents to match the IR-corrected and equilibrium potential-corrected overpotentials.

### Series resistance correction

Uncompensated resistance (*R*_u_) was determined from potentiostatic electrochemical impedance spectroscopy at open circuit potential and remained stable across the applied potential range, indicating that it primarily originated from the electrolyte. The impedance data at open circuit and reaction potentials were fitted to the equivalent electric circuit shown in Supplementary Fig. 25. No dynamic IR compensation was applied during the measurements to avoid potential artefacts such as signal feedback or resonance effects, especially at high current densities. All potentials reported are corrected post-measurement using the measured *R*_u_ value (Supplementary Fig. 26). Importantly, the ohmic drop correction had little impact on the final kinetic maps (Supplementary Fig. 13).

### ECSA

The ECSA was estimated via cyclic voltammograms between 0.96 V and 1.04 V (non-Faradaic region) at different scan rates: 20 mV s^−1^, 50 mV s^−1^, 100 mV s^−1^, 150 mV s^−1^ and 200 mV s^−1^. Then, the ECSA was calculated from ${{\mathrm{ECSA}}}={C}_{{dl}}\times {C}_{s}^{-1}.$, where *C*_*x*_ is the specific capacitance of the sample; a common value of 0.04 mC cm^−2^ was used^75^. Note that the ECSA determined at non-Faradic potentials can be seen only as the active area prone to convert into active sites during the OER, not the bias-dependent active area, which remains hidden in such an analysis. The values of the ECSA can be found in Supplementary Table 3.

### Arrhenius analysis

The activation energy, *E*_A_(*η*), and pre-exponential factor, log(*A*(*η*)), are extracted via Arrhenius analysis of temperature-dependent, multistep, chronoamperometry experiments. At low current densities, changes in [OH^−^] in alkali (or [H_2_O] in acid) are negligible. Furthermore, the reverse oxygen reduction reaction currents are negligible. Conversely, the temperature dependence of the current ${j}_{{{\mathrm{OER}}}}=\left[{\mathrm{O{H}}}^{-}\right]{k}_{{{\mathrm{OER}}}}$ with the rate constant, *k*_OER_, informs directly on:

1 $${k}_{{{\mathrm{OER}}}}=A{(\eta )e}^{\frac{-{E}_{A}(\eta )}{{RT}}}$$

for a first-order reaction, with temperature *T* and gas constant *R*.

The natural logarithm of the averaged steady-state current density was plotted against the reciprocal absolute temperature and linear regression was performed:

2 $${{\mathrm{ln}}(j}(\eta ))=\mathrm{ln}(A(\eta ))-\left(\frac{{E}_{A}(\eta )}{R}\right)\left(\frac{1}{T}\right)$$

For each overpotential (corrected by the temperature dependence of the equilibrium potential, see earlier), the linear slope and *y*-axis intercept at infinite temperatures, ${T}^{-1}\to 0$, were extracted to calculate *E*_A_(*η*) and log(*A*(*η*)), respectively. Natural logarithms were converted to base 10 ones and standard linear regression errors for the slope and *y*-axis intercept were used as error bars. In addition, the coefficient of determination (*R*^2^) of the Arrhenius linear regression was used as a measure of the degree of linearity for different potentials within different experiments.

### X-ray absorption spectroscopy

In situ XAS measurements were carried out at the SOLEIL beamline of the SAMBA synchrotron in fluorescence mode at the Ni–K edge (8,333 eV). An energy-selective, 32-channel Ge detector was used for fluorescence signal collection and a Si(220) monochromator for energy selection. Samples were spray coated on carbon paper and mounted as a working electrode in our in-house-built, single-compartment, electrochemical cell^76^. A Pt mesh was used as a counter-electrode. A leak-free Ag or AgCl electrode was used as a reference. During the experiments, Ar gas was continuously bubbled through the electrolyte, which was also circulated using a peristaltic pump. The applied potential was controlled by a BioLogic potentiostat. Spectra alignment, background subtraction and normalization of the collected XAS data, as well as LCA of the extracted XANES spectra, were performed using a series of in-house-built Wolfram Mathematica scripts. For the LCA as reference spectrum, we used the spectra for the as-prepared sample measured in air and the spectrum for γ-NiOOH measured during the same beamtime.

A γ-NiOOH reference sample was prepared, by first electrodepositing Ni from a Ni acetate solution (0.1 M NaNO_3_ + 5 mM Ni acetate) on a Pt electrode for 2 min at −0.8 V_RHE_. The electrolyte was then removed, the sample rinsed with water and then exposed to 0.1 M KOH electrolyte. After activation by two cyclic voltammetry scans between 0.6 V and 1.8 V_RHE_ (25 mV s^−1^ scan rate), the sample was oxidized at 1.8 V_RHE_ until no further changes were visible in XAS.

To quantify the average oxidation state, we relied on LCA of the collected XANES spectra (Supplementary Figs. 18 and 19). Specifically, we noted that all XANES spectra collected to a good accuracy can be expressed as a linear combination of spectra for the respective as-prepared catalyst (which is dominated by Ni^2+^ species) and the spectrum for the electrochemically prepared γ-NiOOH reference, where the average oxidation state of Ni is commonly assigned as +3.6 (ref. ^77^).

### X-ray diffraction

Operando, time-resolved HE-XRD experiments were conducted at the beamline ID31 of the European Synchrotron Radiation Facility (ESRF)^78^. The XRD patterns were recorded at an X-ray energy of 75 keV (*λ* = 0.1653 Å (0.01653 nm)) using a Dectris Pilatus X CdTe 2-M detector at an integration time of 1 s. The working distance of the detector was set as 0.7515 m and calibrated by collecting the diffraction pattern of a CeO_2_ standard. Masking and radial integration of the detector images were performed using the software package pyFAI^79^. Time-resolved diffraction patterns were analysed by sequential Rietveld refinement using the Bruker-AXS software package TOPAS, taking into account the zero error, as well as the fit parameters scale factor, lattice parameters and Lorentzian size-induced peak broadening, as detailed later. The β-Ni(OH)_2_ and γ-NiOOH phases were refined using R-3m space groups based on crystallographic information files from ref. ^74^.

Electrochemical XRD was carried out in a homemade electrochemical flow cell and the potential was controlled by a BioLogic SP-300 potentiostat. A leak-free Ag or AgCl reference electrode and a Pt mesh counter-electrode were used. The catalyst was spray coated on carbon paper at a catalyst loading of 0.1 mg cm^−2^. Rigorously Fe-free, 0.1 M KOH was circulated using a peristaltic pump and continuously degassed with Ar gas. Potential step experiments were performed between 1.3 V_RHE_ and 1.8 V_RHE_ by increasing the anodic potential by 50 mV. Each potential was held for 2 min.

### Rietveld refinement

Rietveld refinement was performed using the software package Topas (v.6, Bruker-AXS) for the diffractograms of the IrO_*x*_ nanoparticles as well as the operando HE-XRD pattern of the Ni(OH)_2_ and NiFe LDH samples. In the former case, the rutile-like IrO_2_ phase was refined using the space group P4_2_/mnm and the metallic Ir with an Fm-3m. The zero error sample displacement has been considered, as well as the lattice parameter, scale factor and size-induced Lorentzian and Gaussian combination broadening used as fit parameters. In the last case, β-Ni(OH)_2_, γ-NiOOH as well as α-NiFe(OH)_2_ and γ-NiFeOOH were refined using the space group R-3m. The zero error, lattice parameter, size-induced broadening and scale factors were used as fit parameters. To ensure a robust fitting procedure, two selected HE-XRD patterns recorded below and above the Ni redox transition were fitted to determine the initial parameters for the sequential fitting of the operando potential step experiments. During the sequential fitting, the size-induced broadening of the Bragg peaks was kept constant and the initial parameters used. The determined structural coherence lengths are 12.6 nm for the α-NiFe(OH)_2_, 5.3 nm for the γ-NiFeOOH, 10.4 nm for the β-Ni(OH)_2_ and 5.7 nm for the γ-NiOOH. To handle the large number of datasets, a customized Python routine was used to perform sequential refinements of the operando HE-XRD datasets.

### TEM measurements

STEM and EDS were performed on a Thermo Fisher Scientific Talos F200x operated at 200 kV. The focussed electron beam was scanned over a specific region and EDS spectra were recorded with a four-quadrant detector (Super-X detector, Thermo Fisher Scientific). HR-STEM images were acquired using a probe-corrected Jeol JEM-ARM 200F equipped with a cold field emission gun operated at 200 kV. Additional high-resolution TEM images were acquired with an image-corrected Thermo Fisher Scientific Titan operated at 300 kV and equipped with a TVIPS XF416 CMOS-based camera.

## Online content

Any methods, additional references, Nature Portfolio reporting summaries, source data, extended data, supplementary information, acknowledgements, peer review information; details of author contributions and competing interests; and statements of data and code availability are available at 10.1038/s41557-025-01932-7.

## Supplementary information

Supplementary Information Supplementary Figs. 1–26 and Tables 1–3.

## Source data

Source Data Fig. 2 Temperature-dependent polarization curves and Arrhenius analysis.

Source Data Fig. 3 Tafel plot, temperature-dependent Tafel slopes and kinetic maps.

Source Data Fig. 4 Impact of Fe incorporation into NiO_*x*_ and calcination temperature for IrO_*x*_ in a kinetic map.

Source Data Fig. 5 Bias-dependent XAS, activation parameters, average Ni oxidation state and extracted operando spectroscopy for IrO_*x*_ from ref. ^67^.

Source Data Fig. 6 Bias-dependent activation parameter, average oxidation state and extracted SFG data from ref. ^72^.

## Data Availability

The data supporting the findings of this study are available within the paper and its Supplementary Information. Source data are provided with this paper. Additional tabulated data for Figs. 2–6 are available via Zenodo at 10.5281/zenodo.16527714 (ref. ^80^). XRD data collected at the ESRF are available in ref. ^78^.
