# Author Correction: Interfacial solvation pre-organizes the transition state of the oxygen evolution reaction

**DOI:** 10.1038/s41557-025-01991-w

**Published:** 2025-10-15

**Authors:** Ricardo Martínez-Hincapié, Janis Timoshenko, Timon Wagner, Eduardo Ortega, Jody Druce, Mariana C. O. Monteiro, Martina Rüscher, Joonbaek Jang, Elif Öykü Alagöz, Samuele Lasagna, Leon Jacobse, Arno Bergmann, Beatriz Roldan Cuenya, Sebastian Z. Oener

**Affiliations:** Department of Interface Science, Fritz-Haber Institute of the Max Planck Society, Berlin, Germany

**Keywords:** Electrocatalysis, Electrochemistry

Correction to: *Nature Chemistry* 10.1038/s41557-025-01932-7, published online 3 September 2025.

In the version of the article initially published, in Fig. 2d the label “10^−2^ mA cm^−2^” should have read “1 mA cm^−2^” and in Fig. 2e, the label “1 mA cm^−2^” should have read “10 mA cm^−2^”. In both panels in Fig. 4, the label “10^−1^ mA cm^−2^” should have read “10 mA cm^−2^”. In Supplementary Fig. 10d, the label “10^−2^ mA cm^−2^” should have read “10^2^ mA cm^−2^”. These corrections have been made to the HTML and PDF versions of the article.

