# Supplementary material for: Interfacial solvation pre-organizes the transition state of the oxygen evolution reaction
Source: Nat Chem. 2025 Sep 3;18(5):835–43. doi: 10.1038/s41557-025-01932-7 (PMC13149036; doi:10.1038/s41557-025-01932-7)
Supplement: Supplementary file 1 — Supplementary Figs. 1–26 and Tables 1–3. [file 41557_2025_1932_MOESM1_ESM.pdf]

nature chemistry

Supplementary information

https://doi.org/10.1038/s41557-025-01932-7

Interfacial solvation pre-organizes the transition state of the oxygen evolution reaction

In the format provided by the authors and unedited

This document contains:

Supplementary Figures 1-26

Supplementary Tables 1-3

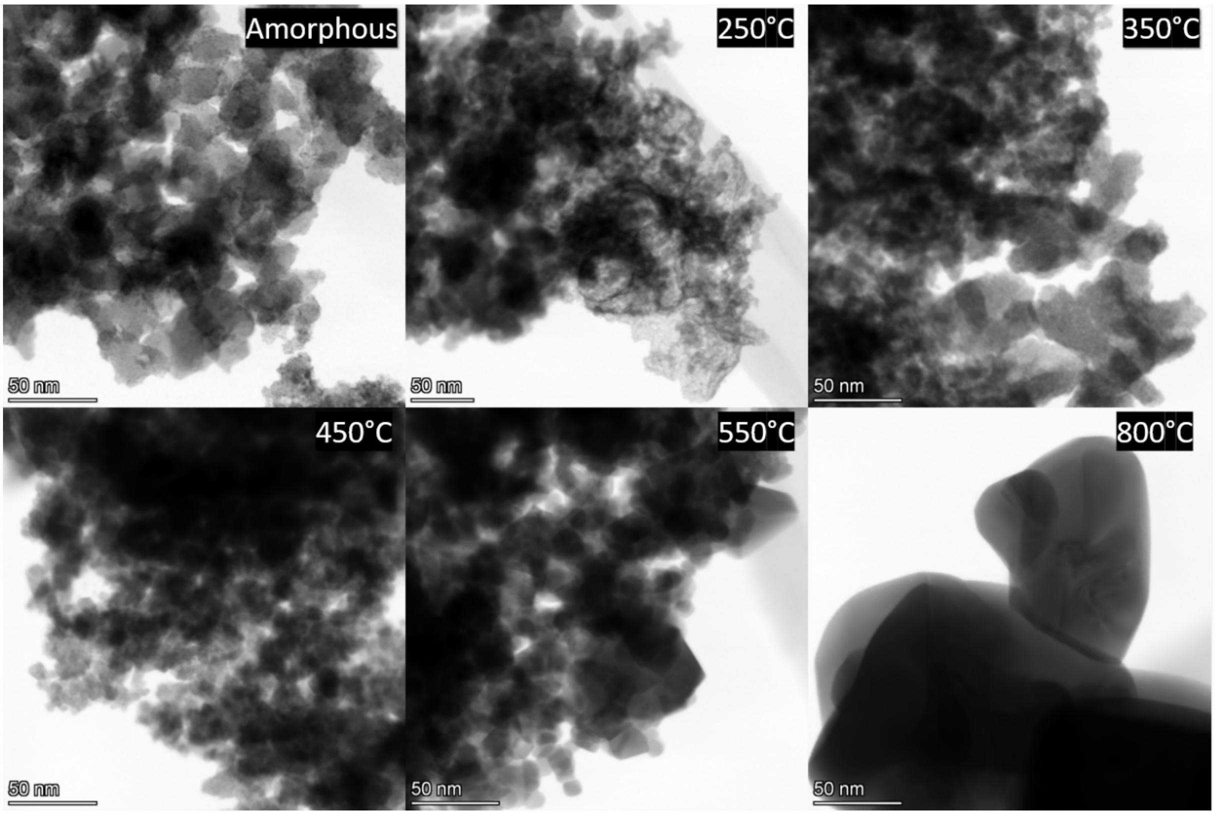

Supplementary Figure 1. Scanning transmission electron microscopy bright-field images (STEM-BF) of IrO_x nanoparticles (NPs) as prepared (amorphous) and after annealing at different temperatures. No catalyst support is present.

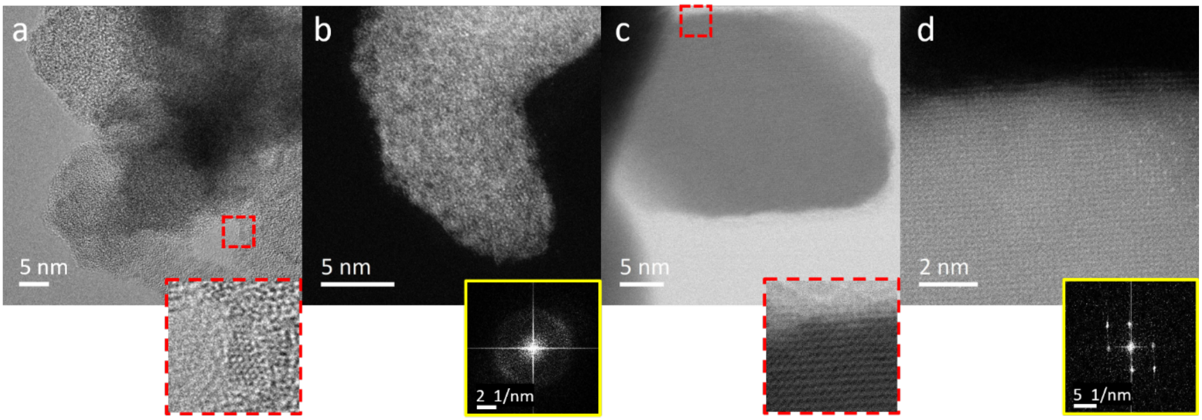

Supplementary Figure 2. High Resolution TEM (HR-TEM) and STEM high angle annular darkfield (HAADF) of (a-b) the initial/amorphous state of the nanocrystalline IrO_x catalyst and (c-d) the state after the 800°C annealing process. Insets show crystallinity either by zooming in (dashed lines) or by means of Fourier transforms (solid lines).

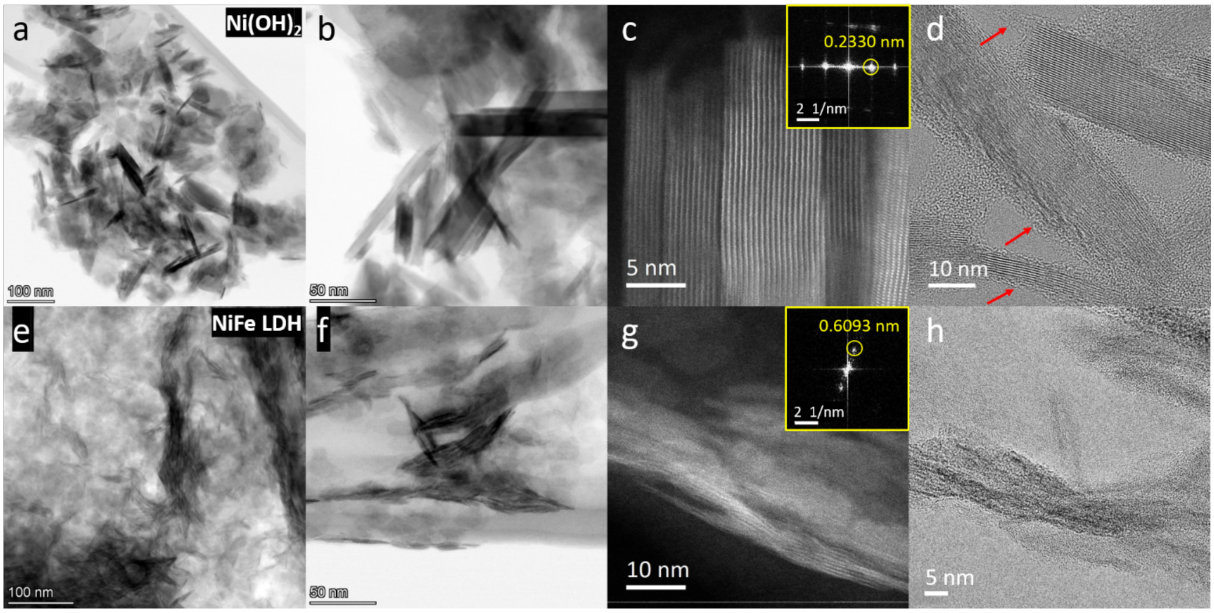

Supplementary Figure 3. STEM-BF, STEM-HAADF and HRTEM images of (a-d) $Ni(OH)_2$ and (e-h) NiFe LDH catalyst before reaction. Insets show the interlayer distance while the presence of amorphous carbon is highlighted on the HRTEM.

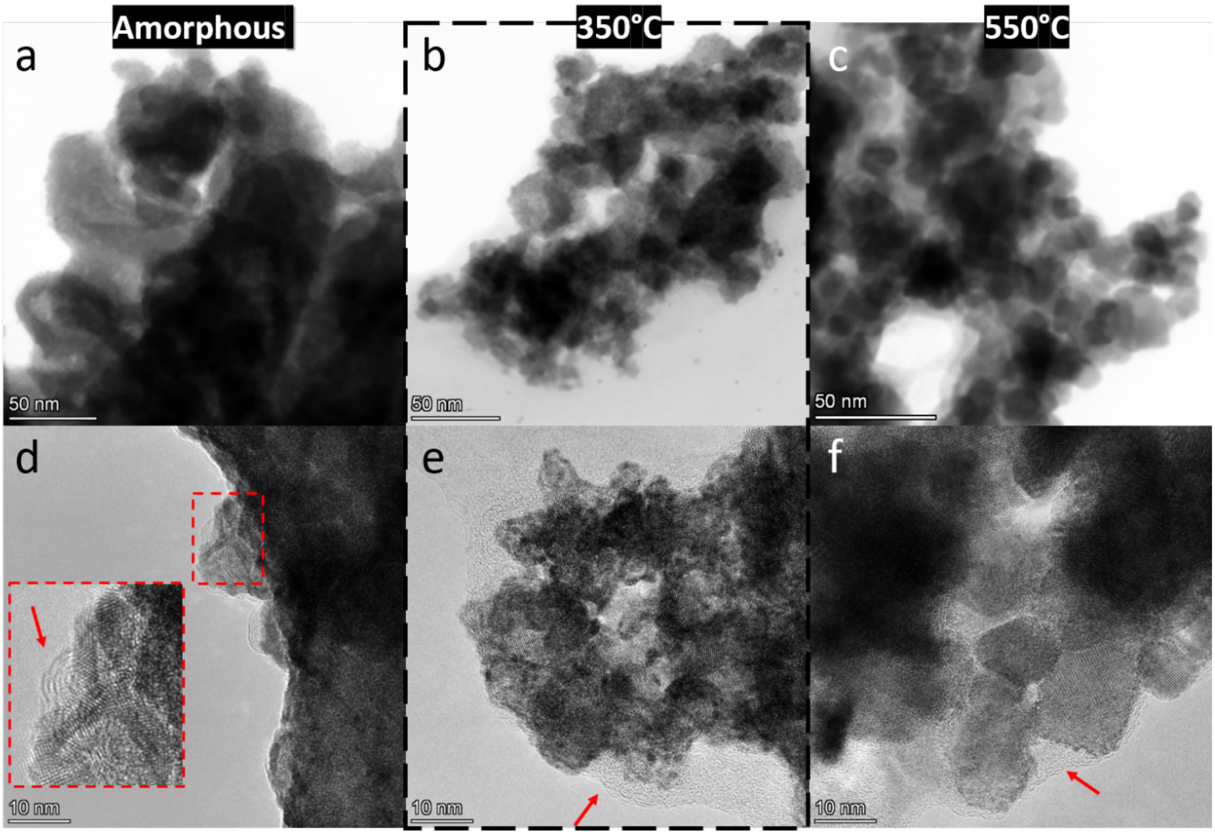

Supplementary Figure 4. (a-c) STEM-BF and (d-f) HRTEM images of selected IrO_x catalysts after reaction. Insets show some degree of crystallinity on the surface of the amorphous sample. Further, the presence of amorphous carbon contamination is highlighted.

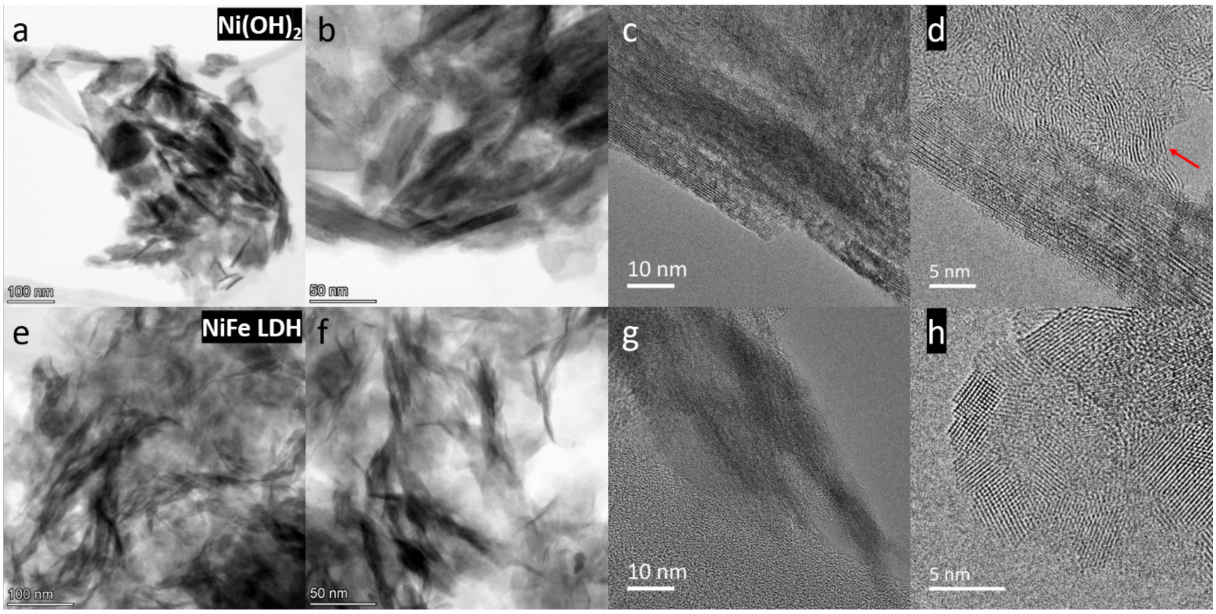

Supplementary Figure 5. STEM-BF and HRTEM images of (a-d) $Ni(OH)_2$ and (e-h) NiFe LDH catalyst after reaction. The presence of graphitic-carbon is highlighted by a red arrow.

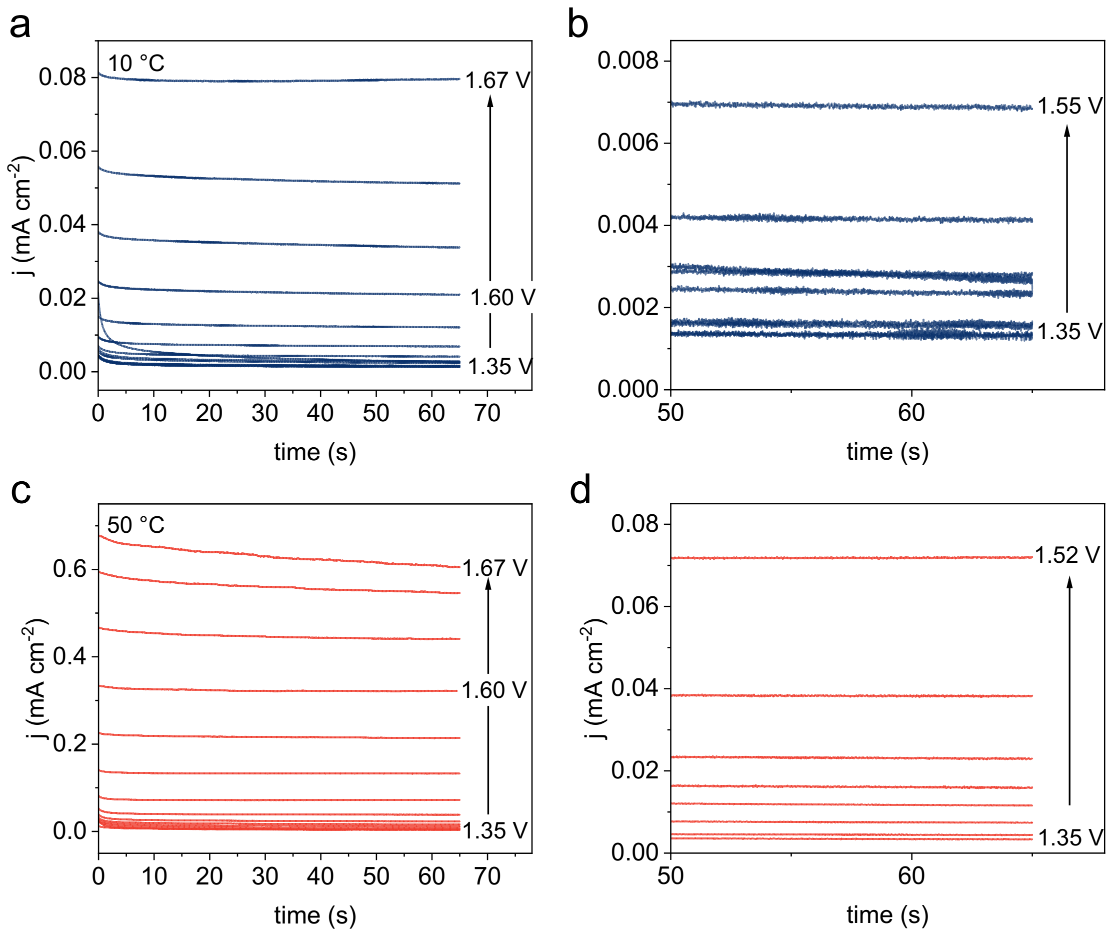

Supplementary Figure 6. Representative chronoamperometries (CA) at different overpotentials in 0.1M KOH for $Ni(OH)_2$. CA's for $Ni(OH)_2$ (a,c) at different overpotentials and different temperatures. Enlarged of the lower potential zone (b,d). The use of CA is preferred over LSV/CV methods to obtain the kinetic data. As the overpotential is increased the noise level also increases, likely due to bubbles on the electrode surface.

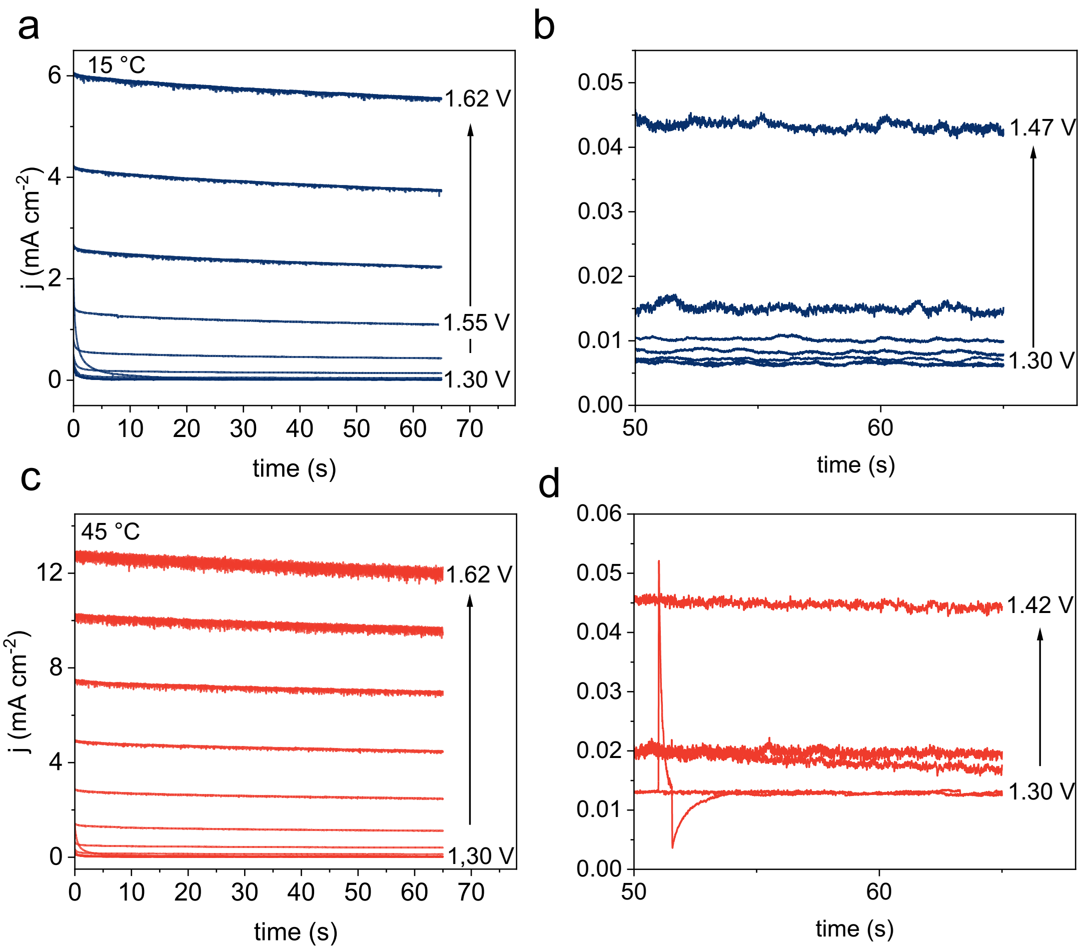

Supplementary Figure 7. Representative chronoamperometries (CA) at different overpotentials in 0.1M H_2SO_4 for IrO_x. CA's for IrO_x (a,c) at different overpotentials and different temperatures. Enlarged of the lower potential zone (b,d). As the overpotential is increased the noise level also increases, likely due to bubbles on the electrode surface.

a		b		c	
η / V	R^2	η / V	R^2	η / V	R^2
0.42	0.985	0.28	0.841	0.35	0.919
0.397	0.976	0.272	0.806	0.334	0.962
0.374	0.952	0.265	0.827	0.318	0.965
0.351	0.946	0.257	0.845	0.302	0.959
0.328	0.959	0.249	0.804	0.285	0.977
0.305	0.978	0.242	0.866	0.269	0.985
0.282	0.994	0.234	0.929	0.253	0.923
0.258	0.998	0.226	0.935	0.237	0.99
0.235	0.992	0.218	0.934	0.221	0.933
0.212	0.994	0.211	0.972	0.205	0.903
0.189	0.994	0.203	0.812	0.188	0.642
0.166	0.997	0.195	0.421	0.172	0.77
0.143	0.943	0.188	0.112	0.156	0.815
0.12	0.79	0.18	0.106	0.14	0.844

Supplementary Figure 8. Heatmap for R^2 values from the linear Arrhenius analysis of the data displayed in Figure 2. a-c, Arrhenius analysis for $Ni(OH)_2$, NiFe LDH and IrO_x. The R^2 values are generally well above 0.9, ensuring high accuracy of the E_A and A values. However, at very low overpotentials very low values of R^2 are common since the current value is close to the noise level. Also, at very high overpotentials problems associated with mass transport and the bubble accumulation in the electrode surface interferes with the analysis.

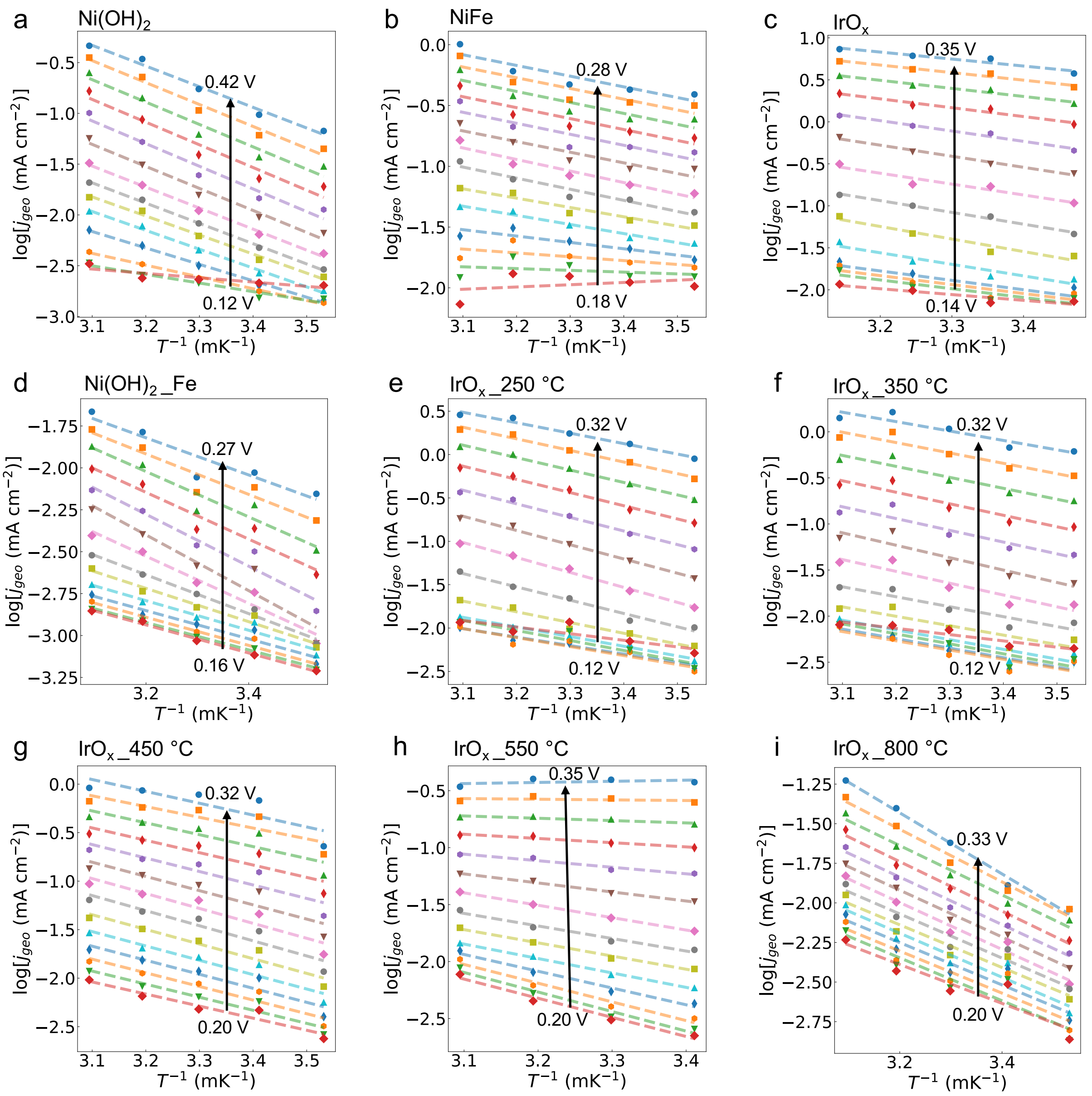

Supplementary Figure 9. Arrhenius analysis of the data displayed in the main manuscript. a-c, Arrhenius analysis for oxygen evolution reaction (OER) on Ni(OH)2, NiFe LDH and IrOx, **d,** on $Ni(OH)_2$ in non-purified 0.1 M KOH. **e-i,** OER on IrOx calcined at different temperatures as indicated. The R2 values are generally well above 0.9, ensuring high accuracy of the EA and A values. However, at very low overpotentials low values of R2 are common, since the current value is close to the noise level. Finally, at high overpotentials, mass transport and bubble accumulation interfere with the analysis. For better traceability, overpotentials shown are uncorrected for the temperature dependence of the equilibrium potential, whereas the data in the main are corrected for the temperature dependence (see details in Methods section).

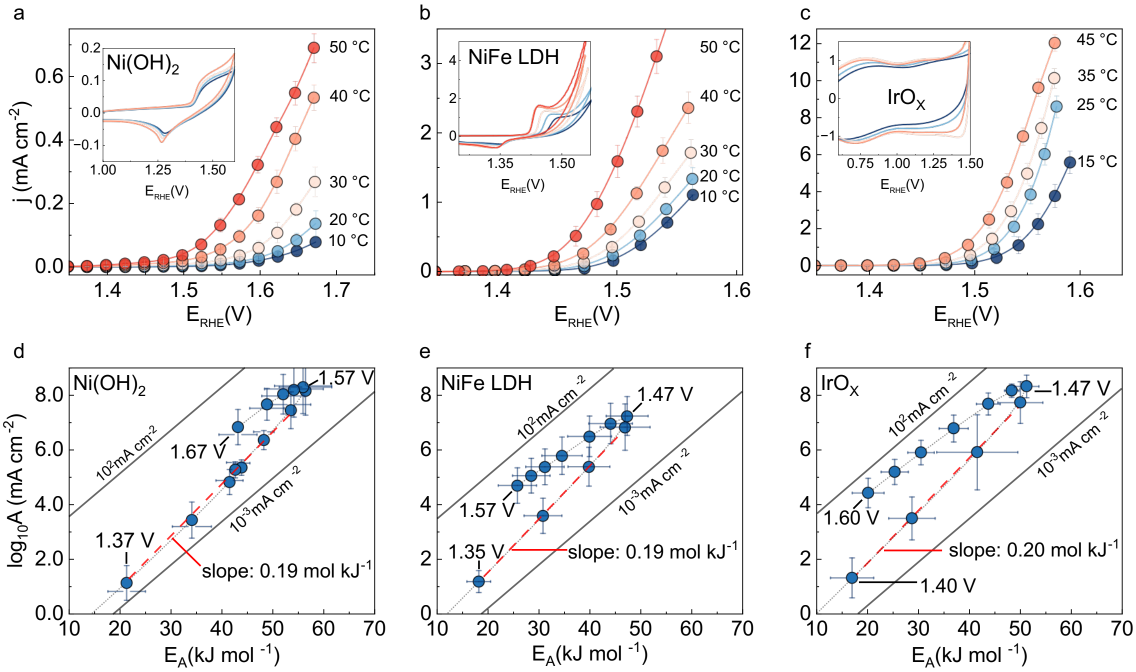

Supplementary Figure 10. Polarization curves and potential dependence on the activation energy and exponential factor without correction of the temperature dependent equilibrium potential. a-c, Characteristic polarization curves for Ni(OH)$_2$ and NiFe LDH in 0.1M Fe-purified KOH and IrOx in 0.1M H_2SO_4, respectively. **d-f** Pre-exponential factor ($log\,A(E_{RHE})$) vs. activation energy ($E_A(E_{RHE})$) for Ni(OH)$_2$, NiFe LDH and IrO$_x$ from panel a-c, respectively. In contrast to the main, here the potential dependent Arrhenius analysis was conducted using the absolute potential, E_{RHE}.

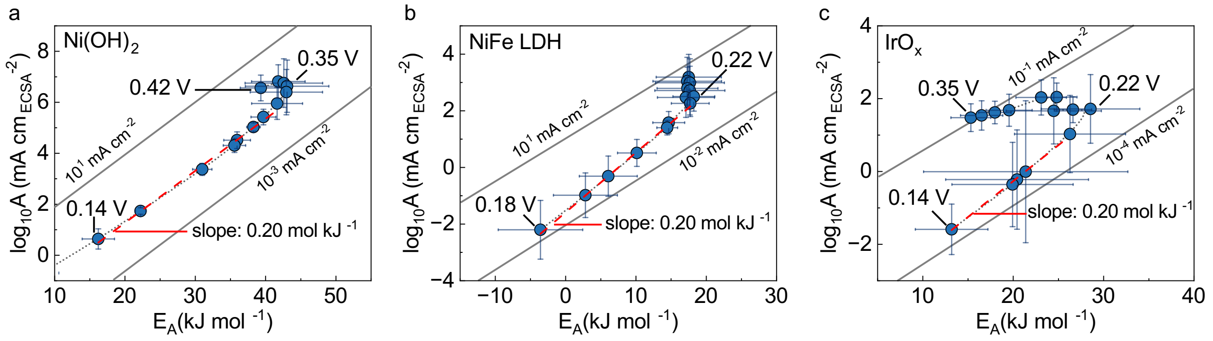

Supplementary Figure 11. Impact of ECSA normalization on the kinetics plots. Overpotential dependent pre-exponential factor ($log\,A(\eta)$) vs. activation energy ($E_A(\eta)$) for $Ni(OH)_2$, NiFe LDH and IrO_x nanoparticles using the ECSA for current normalization. The values of the turning point and activation energy remain constant, while the values of the pre-exponential factor increase. The (over)potentials shown are corrected for the temperature dependence of the equilibrium potential.

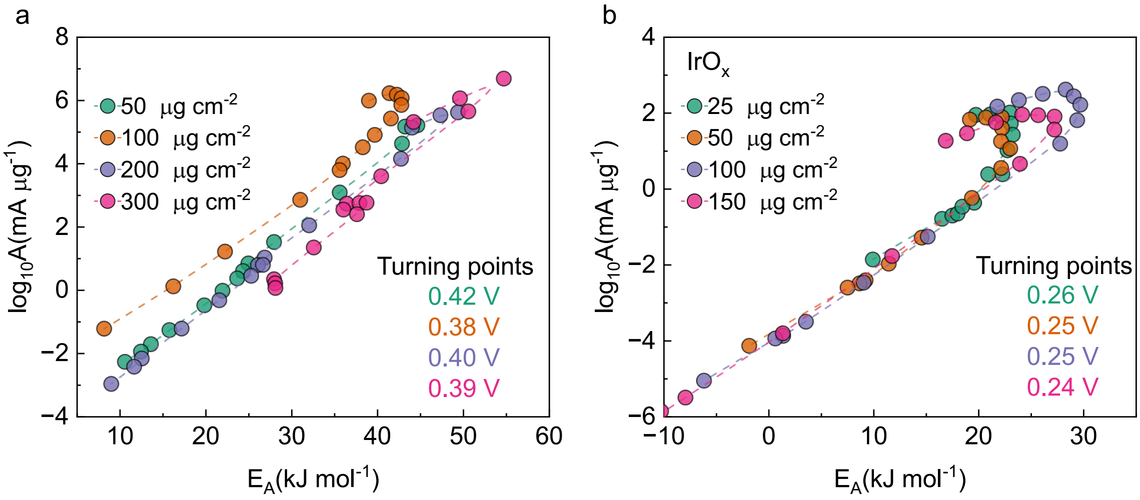

Supplementary Figure 12. Impact of the loading on the kinetics plots. Overpotential dependent pre-exponential factor ($log\,A(\eta)$) vs. activation energy ($E_A(\eta)$) for $Ni(OH)_2$ and IrO_x using different loadings to normalize the current. The data is corrected for the temperature dependence of the equilibrium potential.

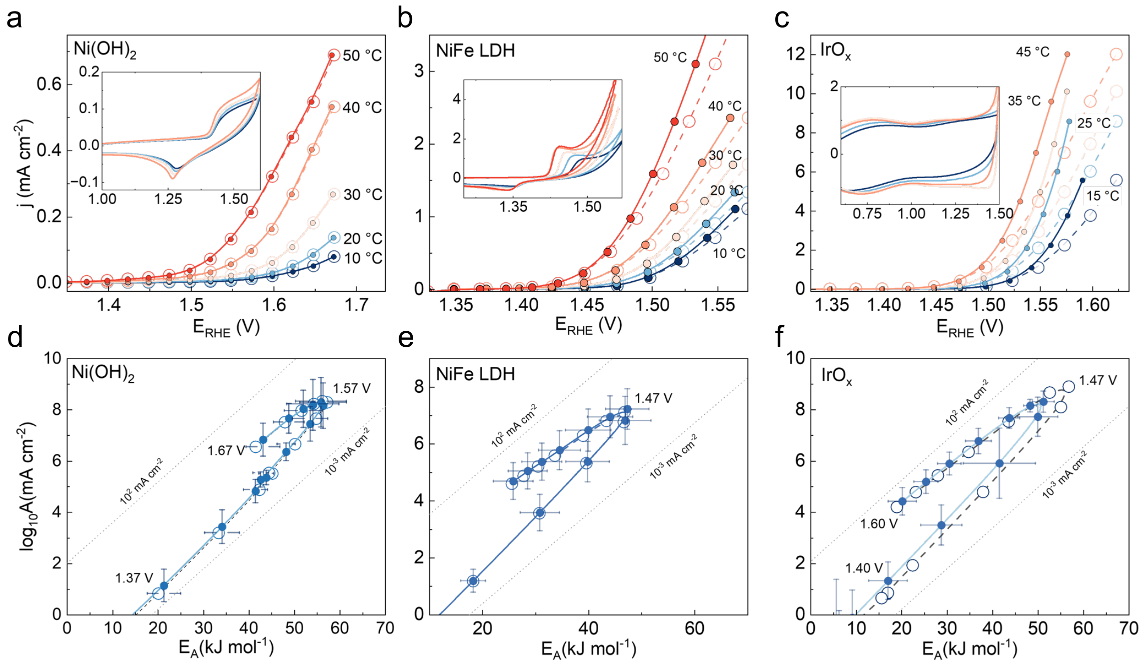

Supplementary Figure 13. Impact of ohmic drop correction on the polarization curves and kinetic plots. 100% IR corrected (filled circles) and un-corrected (open circles). **a-c**, Characteristic polarization curve for $Ni(OH)_2$, NiFe LDH in 0.1M Fe-purified KOH and IrOx in 0.1M H_2SO_4, respectively. **d-f,** Potential (E_{RHE}) dependent pre-exponential factor ($log\,A(E_{RHE})$) vs. activation energy ($E_A(E_{RHE})$) for $Ni(OH)_2$, NiFe LDH and IrO_x from panel a-c, respectively. The ohmic drop correction has a subtle effect for $Ni(OH)_2$ and NiFe LDH as the currents are very low. In contrast, for IrOx, the changes in the polarization curve are notable. However, the turning point remains unchanged. For better traceability of the impact of the Ohmic drop correction, $log\,A(E_{RHE})$ and $E_A(E_{RHE})$ are plotted directly from the as-measured steady-state data, i.e. without correction of the temperature dependent equilibrium potential. In contrast to $log\,A(\eta)$ and $E_A(\eta)$, $log\,A(E_{RHE})$ and $E_A(E_{RHE})$ link the turning potential more closely to the characterization on an RHE scale (e.g. in Figure 5), but obscure the effect of the overpotential (free energy driving force) on the kinetics, which requires accounting for the temperature dependent equilibrium potential (Methods).

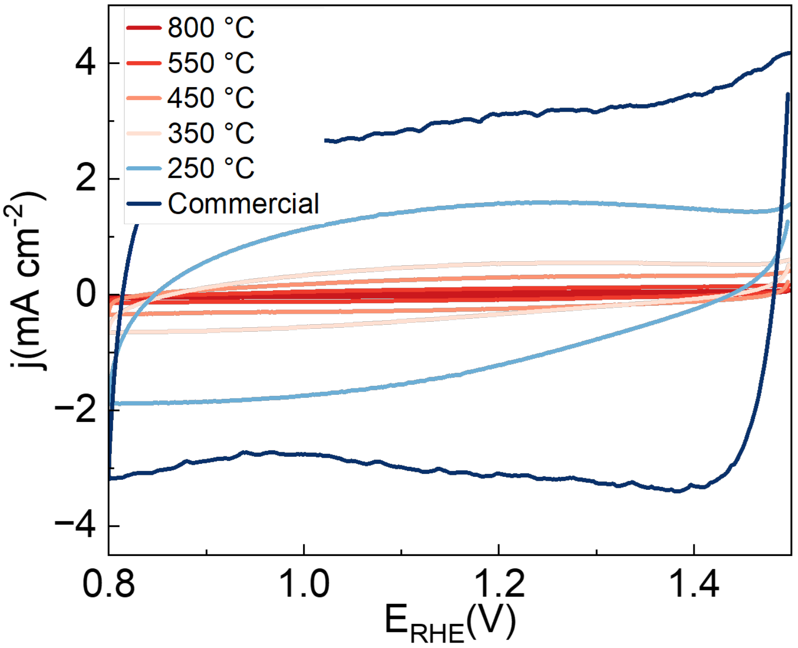

Supplementary Figure 14. Cyclic voltammograms of IrO_X nanoparticles calcined at different temperatures. IrO_X NPs were calcinated at the indicated temperatures for 6 hours in an atmosphere of synthetic air. Cyclic voltammograms of the different IrO_X NPs with same loading (50 µg/cm^2) obtained at a scan rate of 50 mV s^{-1} in 0.1M H_2SO_4. The current is normalized by the geometric area (0.196 cm2). The CVs revel that an increasing calcination temperature leads to a reduction of the capacitance in the voltammetry as result of an increase in the nanoparticle size and reduction of surface area. Importantly, the turning point in the compensation plots does *not* depend on the electrochemical active surface area.

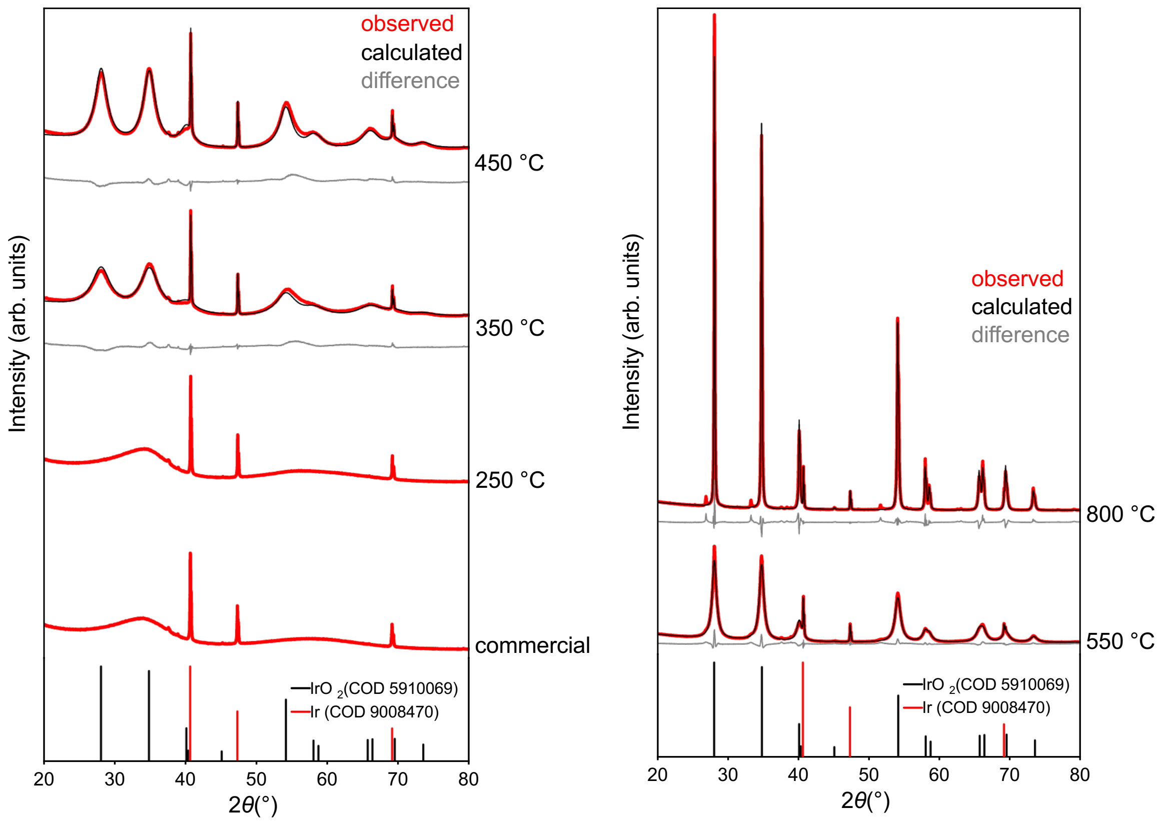

Supplementary Figure 15. X-ray diffraction patterns of the powders as-received and after calcination at temperatures indicated (6 h in synthetic air atmosphere). As the calcination temperature is increased the powder start to show more symmetric and sharper reflections. The presence of broader peaks in middle temperature suggest the presence of small crystalline domains. According to the Rietveld refinement, the size of these domains is around 2-4 nm. See Supplementary Table 2 for more information.

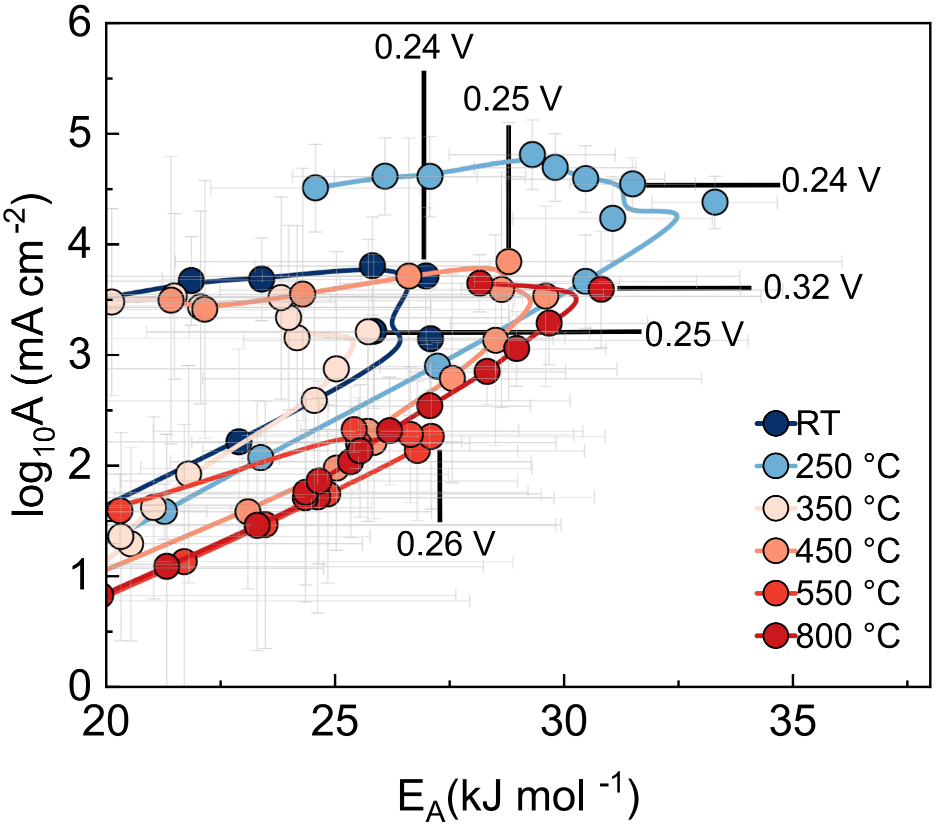

Supplementary Figure 16. Effect of calcination temperature on the kinetic plot. Overpotential dependent pre-exponential factor ($log\,A(\eta)$) vs. activation energy ($E_A(\eta)$) for iridium nanoparticles calcined at different temperatures. The overpotentials shown are corrected for the temperature dependence of the equilibrium potential.

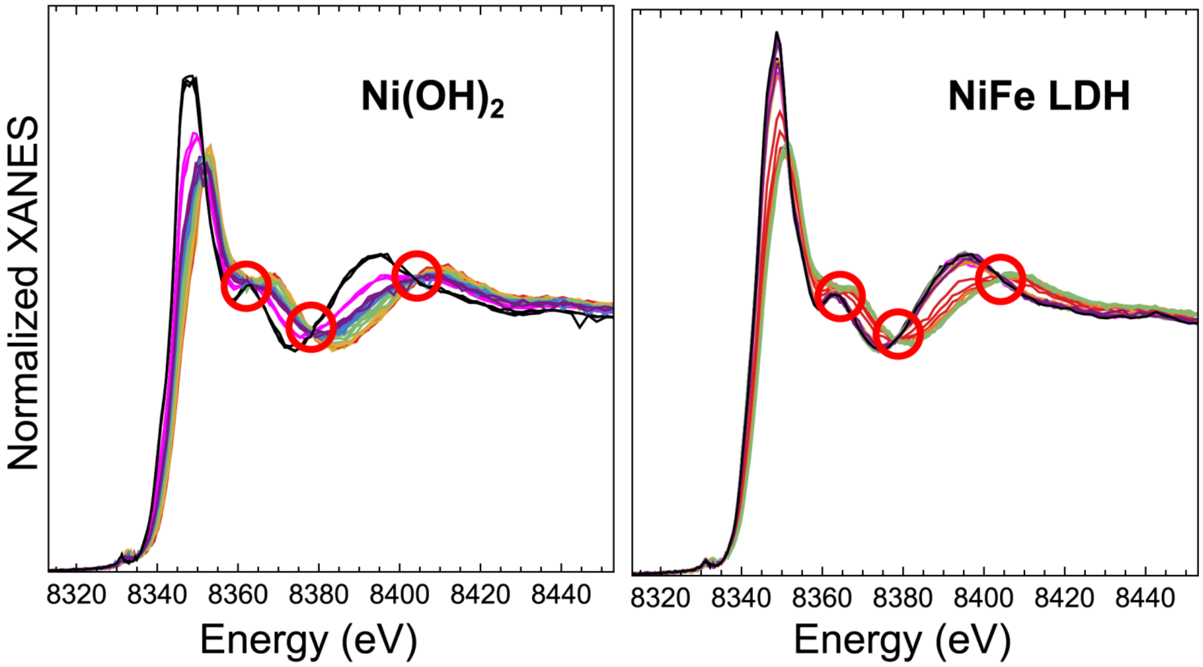

Supplementary Figure 17. Potential dependent Ni K-edge XANES data for $Ni(OH)_2$ and NiFe LDH. The depicted spectra are the same as in Figure 5 in the main text, but are shown without vertical offset. Different line colors correspond to different potentials applied. The same colors are used here as in Figure 5 in the main text. The red circles mark isosbestic points, which are a strong indication that all the variations between spectra can be well represented by a change in a single latent parameter – average Ni oxidation state.

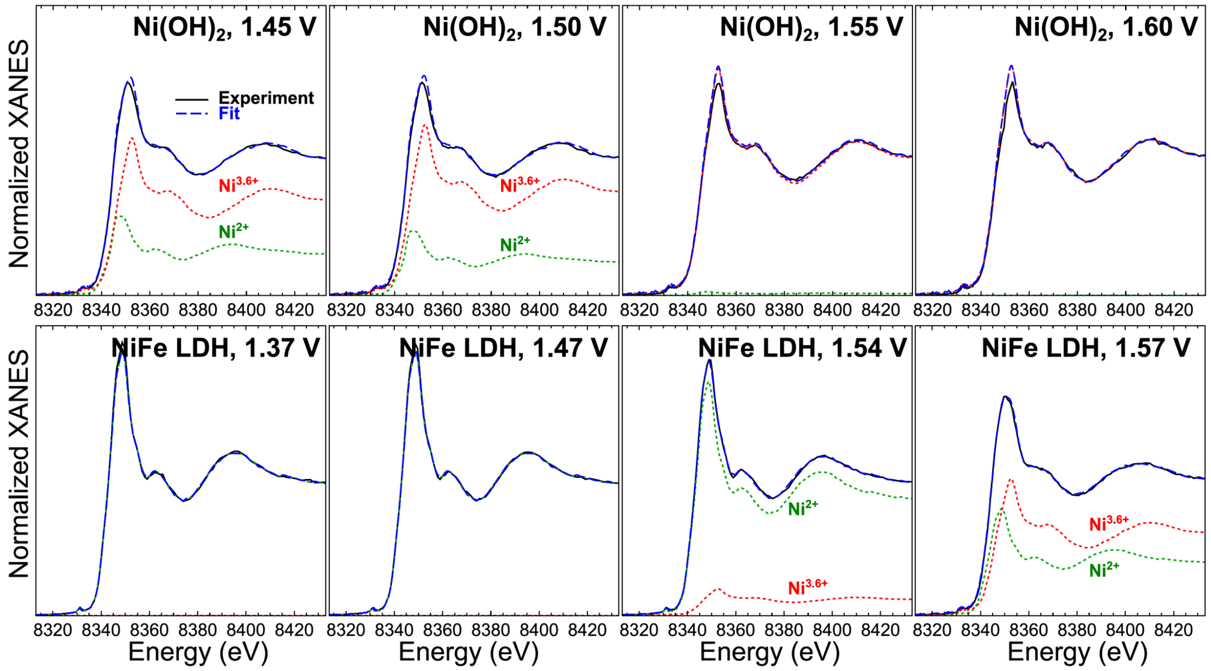

Supplementary Figure 18. Representative examples of linear combination analysis of Ni K-edge X-ray absorption near-edge spectra (LCA-XANES) for $Ni(OH)_2$ and NiFe LDH catalysts. LCA fitting is performed using two species. The spectrum collected for the respective as-prepared catalyst in air is used as a reference for Ni^{2+} species. The spectrum for the electrochemically prepared γ-NiOOH is used as a reference for Ni species with the formal oxidation state of 3.6+. Reference spectra, weighted by their corresponding contribution to the linear combination, are shown as green and red dashed lines.

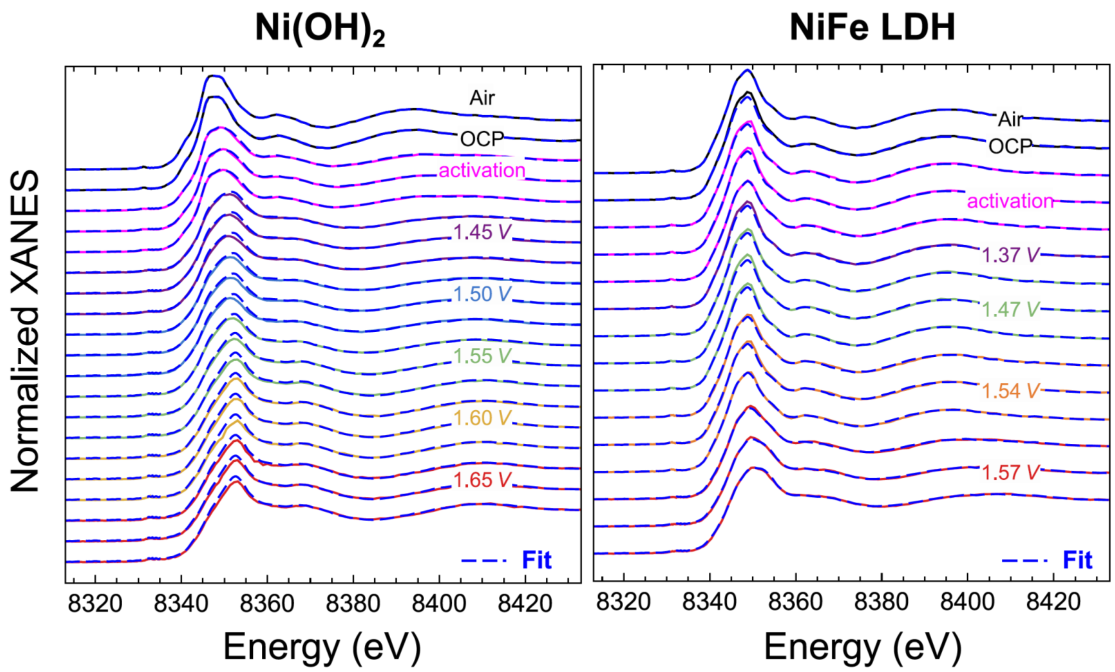

Supplementary Figure 19. Results of linear combination analysis of Ni K-edge X-ray absorption near-edge spectra (LCA-XANES) for $Ni(OH)_2$ and NiFe LDH catalysts. LCA fitting is performed using two species. The spectrum collected for the respective as-prepared catalyst in air is used as a reference for Ni^{2+} species. The spectrum for the electrochemically prepared γ-NiOOH is used as a reference for Ni species with formal oxidation state of 3.6+. Spectra are shifted vertically for clarity.

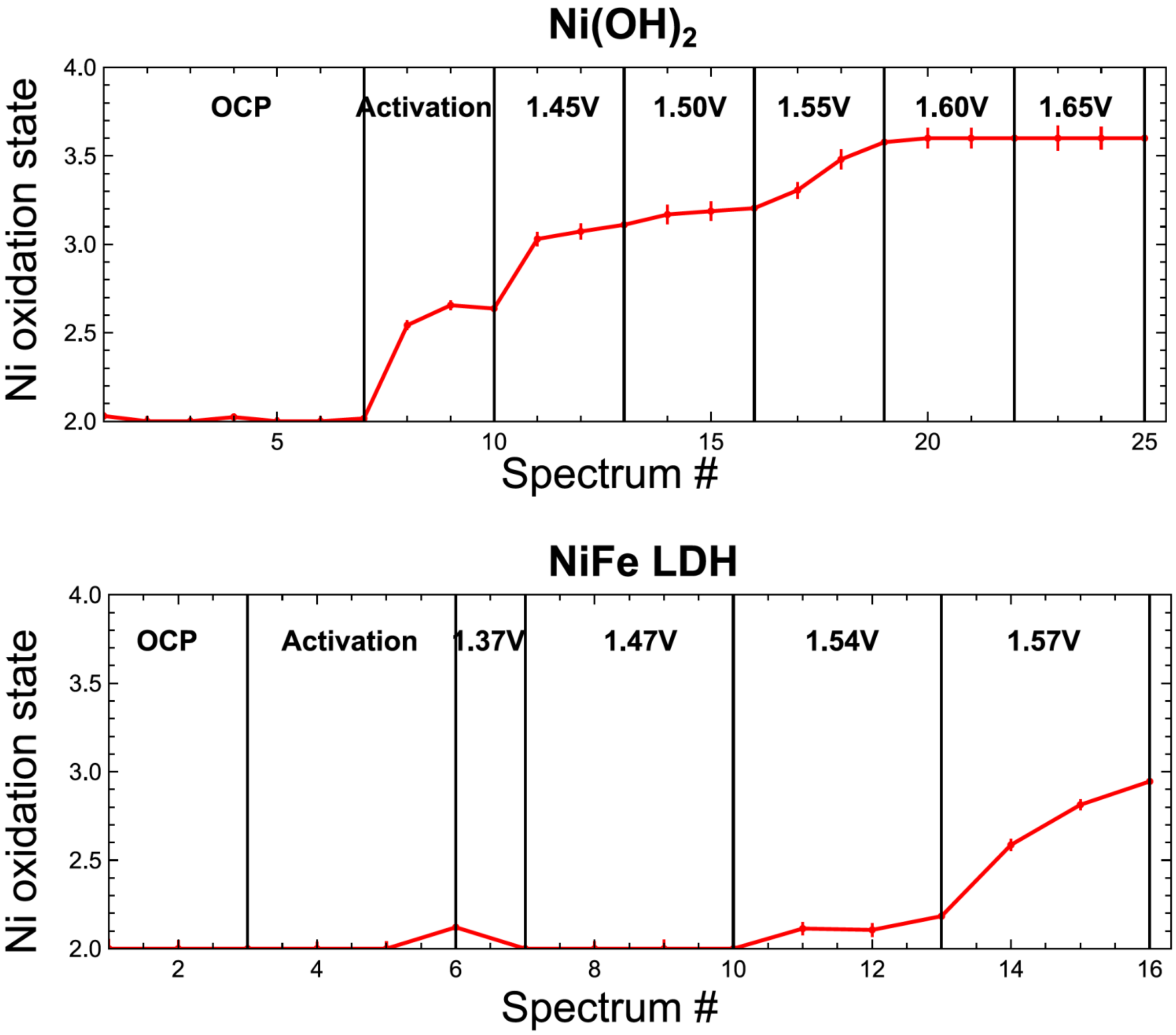

Supplementary Figure 20. Average Ni oxidation state as obtained from the LCA-XANES fits for $Ni(OH)_2$ and NiFe LDH catalysts. Results for individual spectra collected at different potentials.

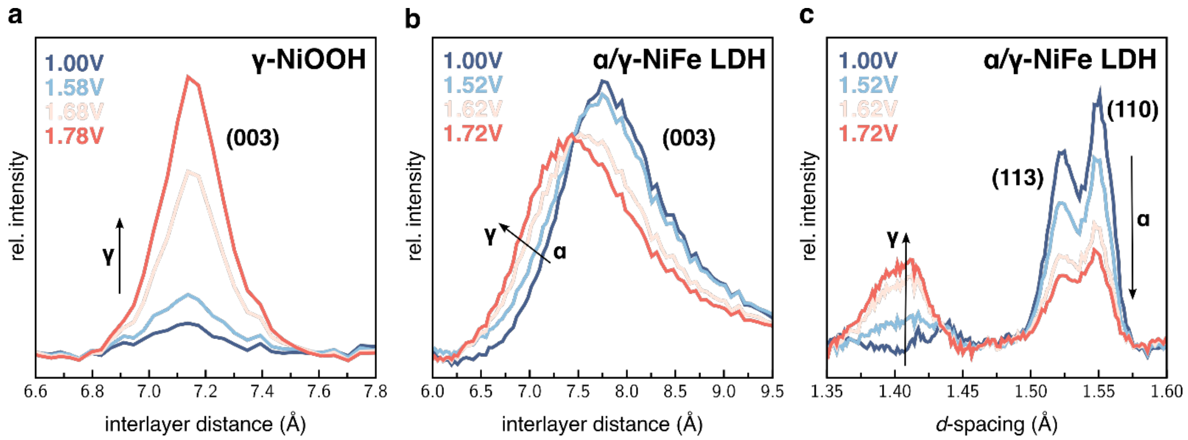

Supplementary Figure 21. Examples of bias dependent high energy X-ray diffraction (HE-XRD). **a,** HE-XRD for γ-NiOOH and, **b-c,** α/γ -$Ni_{1-x}Fe_x(OH)_2$.

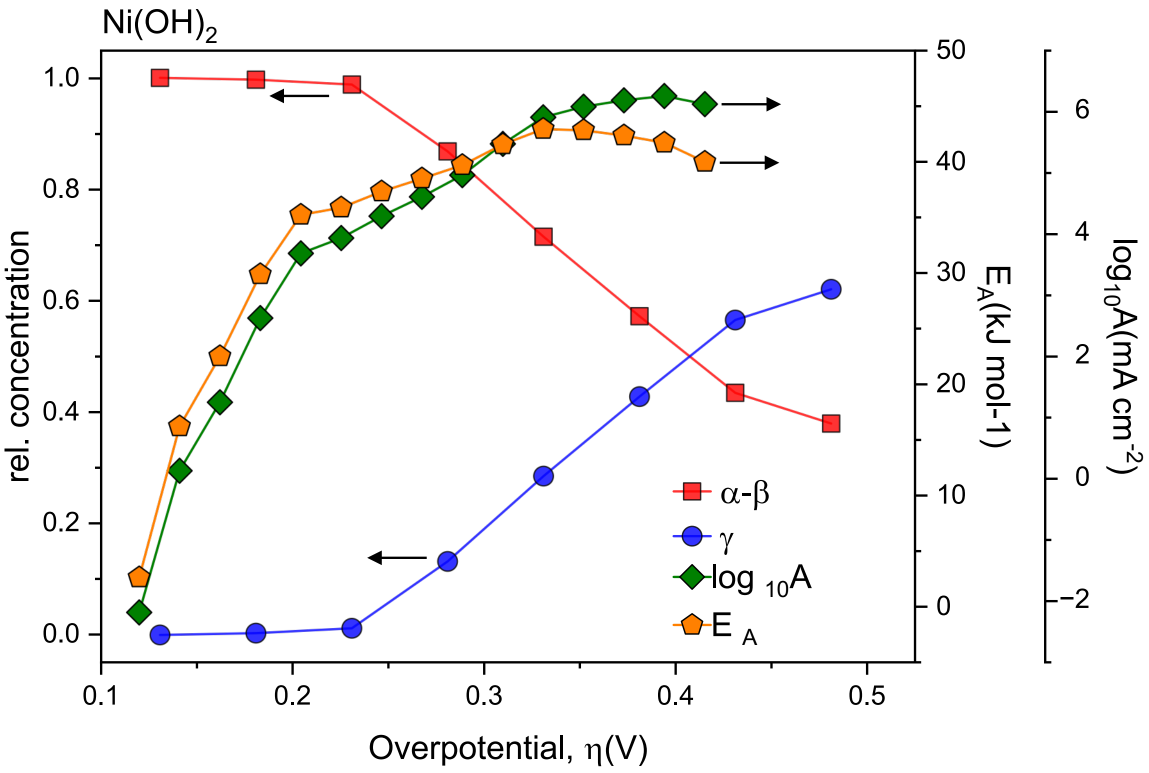

Supplementary Figure 22. Phase and Kinetic Evolution for $Ni(OH)_2$. Correlation of activation parameters with the relative concentrations of different crystal phases obtained from Rietveld refinement. The activation energy, E_A, and pre-exponential factor, *log A*, start to decrease during the emergence of the (frustrated) phase transition between the α/β and γ phases. Prior to this, the increasing E_A closely correlates with increasing $Ni^{3.6+}$ species obtained via X-Ray absorption spectroscopy (see Fig. 5 in main manuscript).

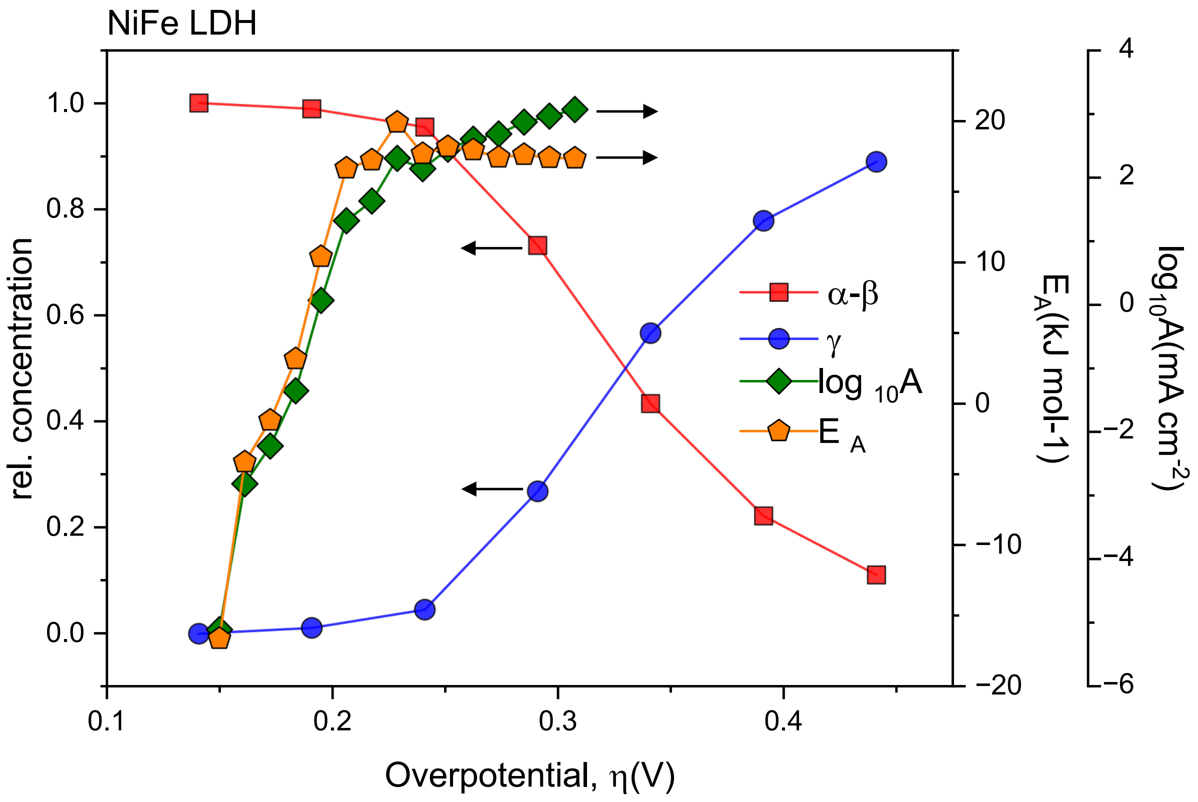

Supplementary Figure 23. Phase and Kinetic Evolution for NiFe LDH Correlation of activation parameters with relative concentrations of different crystal phases obtained from Rietveld refinement. As for $Ni(OH)_2$, the activation energy, E_A, and pre-exponential factor, *log A*, start to decrease right during the emergence of the (frustrated) phase transition between the α/β and γ phases. Prior to this, the increasing E_A correlates with increasing $Ni^{3.6+}$ species obtained via X-Ray absorption spectroscopy (see Fig. 5 in main manuscript).

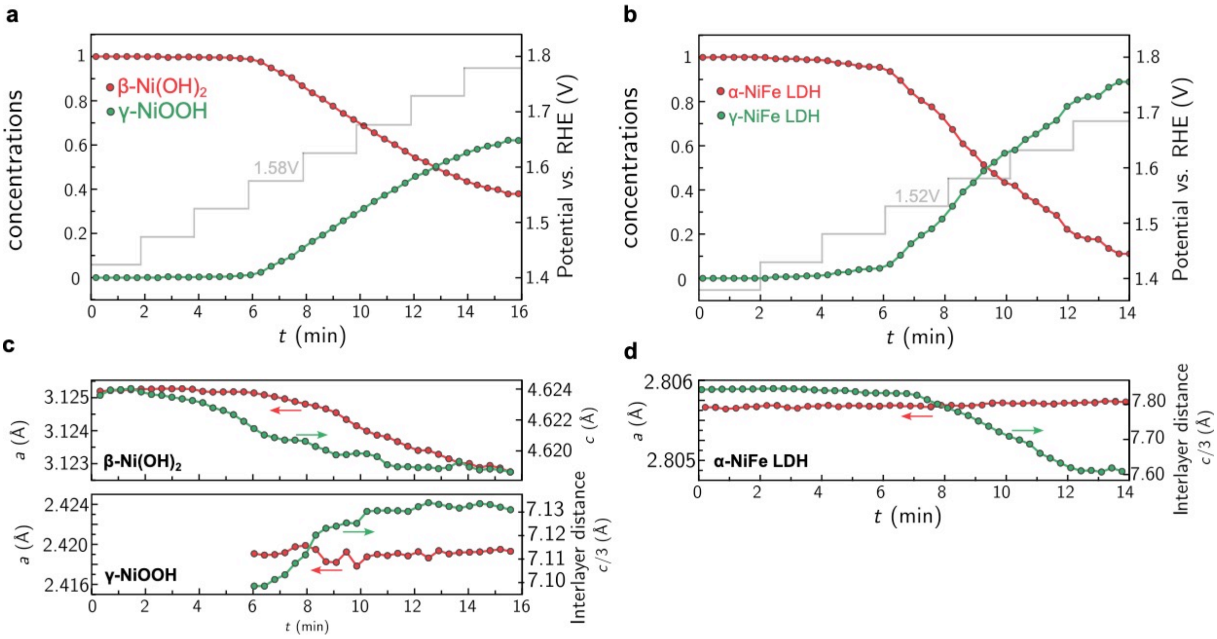

Supplementary Figure 24. *Operando* HE-XRD for Ni-based OER catalysts. **a**, potential-dependent concentrations of crystalline domains of β-$Ni(OH)_2$ and **γ**-NiOOH and **b,** concentrations of α-NiFe LDH and **γ**-NiFe LDH during anodic potential step experiments. **c,** Lattice parameters (*a*, *c*) of $Ni(OH)_2$ and **γ**-NiOOH obtained from sequential Rietveld refinement. **d,** Lattice parameters (*a, c*) of α-NiFe LDH. During Rietveld refinement, the lattice constants of the **γ**-NiFe LDH were held constant.

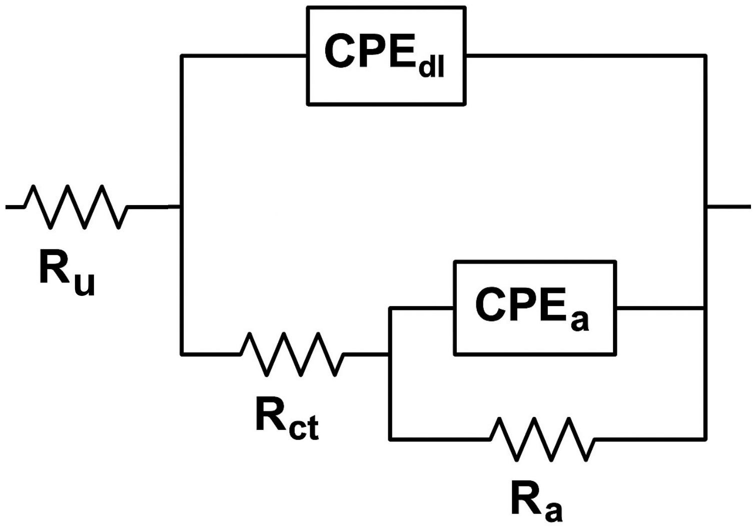

Supplementary Figure 25. Equivalent circuit used to fit the impedance data. The subscripts used for the resistances (R) and constant phase elements (CPE) represent the following: *u* for uncompensated resistance in the electrolyte, *ct* for charge transfer (Faradaic process), *dl* for double layer, and *a* for adsorbates at OER potentials (OER intermediates).

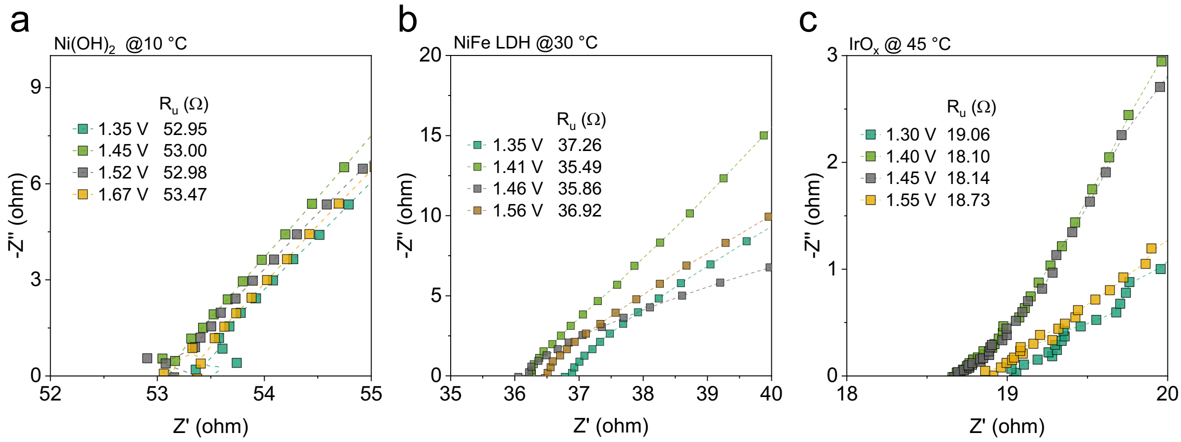

Supplementary Figure 26. Nyquist plots obtained from potentiostatic electrochemical impedance spectroscopy (EIS) at various applied overpotentials for three different OER catalysts: (a) $Ni(OH)_2$, (b) NiFe LDH, and (c) IrO_x. In all cases the uncompensated resistance (R_u) remains nearly constant across the potential range studied. This consistency suggests that R_u is predominantly determined by the electrolyte resistance and is minimally affected by catalyst phase transitions or variations in applied potential.

Supplementary Table 1. STEM EDS semi-quantitative analysis of IrO_x before and after reaction.

		At. % - O	At. % - Ir	Wt. % - O	Wt. % - Ir
Before	Amorph.	74.2 ± 2.1	25.8 ± 2.1	19.3 ± 1.7	80.7 ± 1.7
	250 °C	63.1 ± 2.5	36.9 ± 2.5	12.4 ± 1.1	87.6 ± 1.1
	350 °C	61.5 ± 2.6	38.5 ± 2.6	11.8 ± 1.1	88.2 ± 1.1
	450 °C	63.5 ± 2.5	36.5 ± 2.5	12.6 ± 1.2	87.4 ± 1.2
	550 °C	62.7 ± 2.5	37.3 ± 2.5	12.3 ± 1.1	87.7 ± 1.1
	800 °C	61.0 ± 2.6	40.0 ± 2.6	11.5 ± 1.1	88.5 ± 1.1
After	Amorph.	68.9 ± 2.3	31.1 ± 2.3	15.6 ± 1.4	84.4 ± 1.4
	350 °C	65.0 ± 2.4	35.0 ± 2.4	13.4 ± 1.3	86.6 ± 1.3
	550 °C	62.9 ± 2.5	37.1 ± 2.5	12.3 ± 1.1	87.7 ± 1.1

Supplementary Table 2. Rietveld-refined phase composition and crystallite size estimates (Lvol-FWHM) of Ir-based catalysts subjected to various calcination temperatures.

**Calcination Temperature (°C)**	**IrO_2 (wt%)**	**Ir (wt%)**	**Crystallite Size IrO_2 (nm)**	**Crystallite Size Ir (nm)**
350°C	96.1 ± 0.4	3.9 ± 0.4	2.6 ± 0.2	120 ± 40
450°C	96.01 ± 0.06	3.99 ± 0.06	4.0 ± 0.2	122 ± 10
550°C	96.09 ± 0.16	3.91 ± 0.16	14.1 ± 0.8	105 ± 19
800°C	97.02 ± 0.07	2.98 ± 0.07	147 ± 6	130 ± 30

Supplementary Table 3. Electrochemically active surface area in cm^2.

Electrode	ECSA (cm^2)
$Ni(OH)_2$	0.06
NiFe LDH	0.08
IrO_x	12.5
